# Dietary D-xylose promotes intestinal health by inducing phage production in *Escherichia coli*

**DOI:** 10.1038/s41522-023-00445-w

**Published:** 2023-10-11

**Authors:** Jie Hu, Yifan Wu, Luyuan Kang, Yisi Liu, Hao Ye, Ran Wang, Jinbiao Zhao, Guolong Zhang, Xilong Li, Junjun Wang, Dandan Han

**Affiliations:** 1 State Key Laboratory of Animal Nutrition and Feeding, College of Animal Science and Technology, China Agricultural University, Beijing, China; 2 Department of Animal Sciences, Wageningen University & Research, NL-6700 AH Wageningen, the Netherlands; 3 Key Laboratory for Control Technology and Standard for Agro-product Safety and Quality, Ministry of Agriculture and Rural Affairs, Jiangsu Key Laboratory for Food Quality and Safety-State Key Laboratory Cultivation Base of Ministry of Science and Technology, Institute of Food Safety and Nutrition, Jiangsu Academy of Agricultural Sciences, Nanjing, China; 4 Department of Animal and Food Sciences, Oklahoma State University, Stillwater, OK USA; 5 Key Laboratory of Feed Biotechnology of Ministry of Agriculture and Rural Affairs, Institute of Feed Research, Chinese Academy of Agricultural Sciences, Beijing, China

**Keywords:** Pathogens, Microbiota

## Abstract

Elimination of specific enteropathogenic microorganisms is critical to gut health. However, the complexity of the gut community makes it challenging to target specific bacterial organisms. Accumulating evidence suggests that various foods can change the abundance of intestinal bacteria by modulating prophage induction. By using pathogenic *Escherichia coli* (*E. coli*) ATCC 25922 as a model in this research, we explored the potential of dietary modulation of prophage induction and subsequent bacterial survival. Among a panel of sugars tested in vitro, D-xylose was shown to efficiently induce prophages in *E. coli* ATCC 25922, which depends, in part, upon the production of D-lactic acid. In an enteric mouse model, prophage induction was found to be further enhanced in response to propionic acid. Dietary D-xylose increased the proportion of Clostridia which converted D-lactic acid to propionic acid. Intestinal propionic acid levels were diminished, following either oral gavage with the dehydrogenase gene (*ldhA*)-deficient *E. coli* ATCC 25922 or depletion of intestinal Clostridia by administration of streptomycin. D-Xylose metabolism and exposure to propionic acid triggered *E. coli* ATCC 25922 SOS response that promoted prophage induction. *E. coli* ATCC 25922 mutant of RecA, a key component of SOS system, exhibited decreased phage production. These findings suggest the potential of using dietary components that can induce prophages as antimicrobial alternatives for disease control and prevention by targeted elimination of harmful gut bacteria.

## Introduction

The animal gastrointestinal tract is an important reservoir to a diverse array of microorganisms that consist of bacteria, fungi, viruses, archaea, and protozoa^1,2^. Most human-associated microbes are bacteria, which are known to affect various aspects of intestinal health^3^. Removing pathogenic microbes is considered to be beneficial for the ecology of commensal microbes^4^. However, due to broad-spectrum antimicrobial effects of most antibiotics and drugs, therapeutic strategies targeting specific bacterial species are still limited. An available strategy is to use phages to eliminate specific strains^5^. While lytic phages have long been used to treat various pathogen infections, constant concerns about safety, stability of phages, bacterial resistance to phages, and the time-consuming process of phage screening hamper the adoption of lytic phages in practice^6^. Furthermore, it is challenging to isolate virulent phages for some bacteria, for example, *Escherichia coli* (*E. coli*) ATCC 25922 and *Clostridium difficile*, considering that no researches on strictly virulent phages have been reported^7,8^.

Prophages, viral DNA that originates from temperate phages have been found in approximately one-half of microbial genomes^9^. Despite the numerical dominance of intestinal prophages, they are seldom used for therapeutic purposes. One of the main issues with employing prophages is the inherent capacity of temperate phages to mediate gene transfer between gut bacteria, which may increase bacterial virulence^10,11^. Several lines of research suggest that a large number of bacteriophages in human gut are derived from prophages instead of virulent phages^12,13^. When the prophages are activated under specific conditions, it can lead to the release of progeny phages in the ecosystem and consequently result in host cell death^14^. Therefore, the dynamic changes of intestinal prophages contribute to altering the composition of gut microbiota. Emerging evidence has indicated that targeted regulation of the intestinal microbiota by modulating prophage induction is a potentially practical path for landscaping gut microbiome^14,15^. Prophage induction in the gut ecosystem is largely driven by the changes of the intestinal environment, which is influenced by diet, antibiotics, oxidative stress, and some bacterial metabolites^16^. Specifically, many commonly consumed foods exhibit species-specific growth inhibition of *Bacteroides thetaiotaomicron*, *Enterococcus faecalis*, and *Staphylococcus aureus* via promoting prophage induction in vitro^14^. However, whether a particular dietary prophage inducer can reduce specific bacterial species in the gut and how diet activates intestinal prophages remain largely unexplored to date.

D-Xylose has gradually been regarded as one of the health-beneficial foods. First, a high fraction of D-xylose can be directly absorbed in the human small intestine and subsequently act as a sucrase inhibitor^17–19^. Second, the remaining D-xylose can be transported to the cecum and colon, in which it undergoes bacterial fermentation, thereby modulating the composition of microbiota. For example, dietary supplementation with D-xylose influences the gastrointestinal bacterial community in the Namaqua rock mouse *Aethomys namaquensis*^20^. Moreover, the induction effect of dietary D-xylose on prophages in the gut symbiont *Lactobacillus reuteri* 6475 has been reported^15^. These observations indicate that D-xylose could probably alter the abundance of gut bacteria through inducing the production of temperate phages.

In this study, *E. coli* ATCC 25922, a foodborne pathogenic strain in which genome encodes type III secretion system effector toxin (Gene locus_tag: DR76_RS29130) and enterotoxin (Gene locus_tag: DR76_RS20935), was selected as a model organism to induce enteritis in mice^21–24^ and investigate effects of different dietary sugars on phage production and bacterial growth. We unraveled that dietary D-xylose could effectively decrease *E. coli* ATCC 25922 survival in the gut through activating prophages and phage production was a consequence of D-xylose metabolism by *E. coli* ATCC 25922 and propionic acid stimulation. These results demonstrate that dietary components that can induce prophages is an adjunctive therapeutic tool for gut health improvement.

## Results

### Identification of prophages in *E. coli* ATCC 25922

The chromosome of *E. coli* ATCC 25922 harbored a total of five prophage-like elements (Supplementary Fig. 1a), of which, three complete prophages were identified: prophage Φ1 (38.2 Kb; *Siphoviridae*), prophage Φ2 (49.3 Kb; *Siphoviridae*), and prophage Φ3 (51.3 Kb; *Siphoviridae*) (Supplementary Table 1). Phage genomes of Φ1, Φ2, and Φ3 contained well-defined phage biology information such as tail, head, envelope, lysis, or integrase characteristics. Importantly, no virulence or antimicrobial resistance genes were reported in prophages Φ1, Φ2, and Φ3 genomes. The prophages Φ4 and Φ5 genomes were incomplete and particularly small in comparison with prophages Φ1, Φ2, and Φ3 (~18.7 Kb versus >38 Kb). Prophage Φ4 only encoded integrase, capsid protein, and head-related protein. Prophage Φ5 almost lacked all modules that were responsible for replication, transcriptional regulation, DNA packaging, head and tail morphogenesis, and lysis. Although incomplete/defective prophages may excise, prophage remnants themselves can no longer produce active phages and lyse host bacteria (Supplementary Fig. 1c and Supplementary Table 2)^25^. Therefore, prophages Φ4 and Φ5 were not analyzed in this study. Consistently, mitomycin C significantly induced cell lysis of *E. coli* ATCC 25922, indicating enhanced lysis induction in lysogens (Supplementary Fig. 1b). We further confirmed the presence of phages Φ1 (1.23 × 10^12^ copies/mL), Φ2 (2.46 × 10^14^ copies/mL), and Φ3 (6.26 × 10^13^ copies/mL) in DNA extracted from precipitated phage particles by RT-qPCR, and agarose gel electrophoresis indeed revealed PCR product of expected size for each prophage (Supplementary Fig. 1c and Supplementary Table 2). Transmission electron microscopy showed three different long-tailed phage particles in bacterial culture supernatants (Supplementary Fig. 1d). Collectively, these independent lines of evidence confirm the presence and induction of three complete prophages in *E. coli* ATCC 25922.

### D-Xylose promotes prophage induction in *E. coli* ATCC 25922

**Fig. 1:**
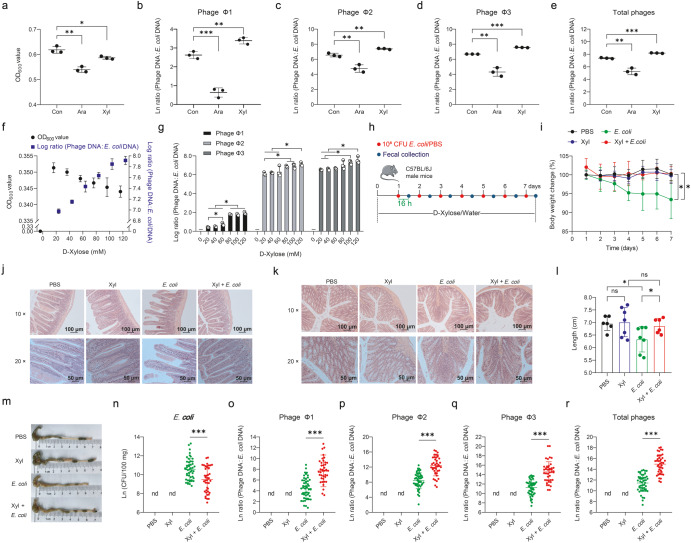
D-Xylose promotes prophage induction in *E. coli* ATCC 25922. *E. coli* ATCC 25922 cell density (**a**) and phage Φ1, Φ2, Φ3, and total phage (sum of phage Φ1, Φ2, and Φ3):*E. coli* DNA ratios (/mL) (**b**–**e**) upon 24 h growth in Lysogeny Broth (LB) medium supplemented with 1% L-arabinose (Ara) or D-xylose (Xyl) (*n* = 3, respectively). **f** Cell density of *E. coli* ATCC 25922 and total phage:*E. coli* DNA ratio (/mL) upon 24 h growth in MOPS minimal medium supplemented with 0, 20, 40, 60, 80, 100, and 120 mM D-xylose (*n* = 3). **g** Phage Φ1, Φ2, and Φ3:*E. coli* DNA ratios (/mL) upon 24 h growth in MOPS minimal medium supplemented with 0, 20, 40, 60, 80, 100, and 120 mM D-xylose (*n* = 3). ‒: No *E. coli* growth. **h** Experimental timeline examining the effect of dietary D-xylose on prophage induction in *E. coli* ATCC 25922. Six/seven mice were used for each treatment group. See “Methods” for more details. Body weight change (**i**), representative H&E staining images (scale bars = 100 μm) of jejunum (**j**) and colon (**k**) and corresponding local high magnification images (Scale bars = 50 μm), colon length (**l**), and representative colon images (**m**). Number of *E. coli* ATCC 25922 (**n**) and phage Φ1, Φ2, Φ3, and total phage:*E. coli* DNA ratios (/100 mg) (**o**–**r**) in feces during an experimental period of 7 days. Each dot in (**i**) represents the mean value of 6/7 mice per group daily. Each dot in (**l**) represents a single data point from a single mouse. Each dot in (**n**–**r**) represents a single data point from a single mouse fecal sample on one day. nd not detectable. Data in (**a**–**e**, **i**, **l**, **n**–**r**) were analyzed using unpaired Studentʼs *t* test. Data in (**g**) were analyzed using one-way ANOVA with Tukey’s test. ns not statistically significant. *P < 0.05, **P < 0.01, ***P < 0.001. Data were expressed as mean ± SD.

To study the impact of several different sugars on prophage induction in *E. coli* ATCC 25922, first, we examined the phage-like particles in the supernatant using transmission electron microscopy. We found that *E. coli* ATCC 25922 produced phages in LB medium containing D-xylose and L-arabinose, while phages were rarely observed in the medium containing glucose, fructose, arabinogalactan, and xylan (Supplementary Fig. 2). Then we further assessed to what extent D-xylose and L-arabinose affect phage production by *E. coli* ATCC 25922. Results showed that supplementation with 1% D-xylose or L-arabinose decreased the cell density (*P* < 0.05) (Fig. 1a). However, D-xylose rather than L-arabinose induced more phages compared with controls as indicated by the increased DNA copies of phages Φ1, Φ2, Φ3, and total phages by up to 2.03-fold (1.44 × 10^11^_Con_ versus 2.93 × 10^11^_Xyl_ copies/mL), 1.97-fold (8.13 × 10^12^ _Con_ versus 1.6 × 10^13^_Xyl_ copies/mL), 2.30-fold (8.36 × 10^12^_Con_ versus 1.92 × 10^13^_Xyl_ copies/mL), and 2.13-fold (1.67 × 10^13^_Con_ versus 3.55 × 10^13^_Xyl_ copies/mL), respectively (*P* < 0.01) (Supplementary Fig. 3), and increased phage Φ1, Φ2, Φ3, and total phage:*E. coli* DNA ratios (*P* < 0.01) (Fig. 1b–e), indicating a possible regulatory role of D-xylose in promoting prophage induction. To better understand the dynamics of prophage induction induced by D-xylose, we performed in vitro experiments within a range of D-xylose concentrations from 20 to 120 mM. With the increasing concentration of D-xylose, phage production (Supplementary Fig. 4) and phage:*E. coli* DNA ratios were gradually increased (Fig. 1f, g), but concomitant with decreased OD_600_ values (Fig. 1f). Therefore, our data have provided the functional evidence that D-xylose can activate prophages in *E. coli* ATCC 25922 in vitro.

We next addressed to whether dietary supplementation of D-xylose affects prophage induction in mice. Throughout the test period, mice were supplemented with D-xylose (1 g/kg BW; 22.5 mg intake per mice/day) in their drinking water. At day 1, mice were received *E. coli* ATCC 25922 for 7 consecutive days (Fig. 1h). Daily, fecal phage DNA from each mice was extracted to perform real-time fluorescence quantitative PCR analyses. Compared with *E. coli* challenge group, dietary D-xylose significantly reduced fecal *E. coli* ATCC 25922 number (*P* < 0.001) (Fig. 1n and Supplementary Fig. 5a), and disease severity as indicated by reduced weight loss (Fig. 1i and Supplementary Fig. 6), serum inflammatory cytokine concentrations (i.e., TNF-α, IL-6, IL-10, and IL-1β) (Supplementary Fig. 7a–d) and liver organ index (the ratio of liver weight to body weight) (Supplementary Fig. 7e, f), as well as improved intestinal development (i.e., improved jejunal morphology shown as neatly arranged villi (Fig. 1j), reduced colonal inflammatory cell infiltration (Fig. 1k), and increased colon length (Fig. 1l, m). While lower phage production was observed during *E. coli* ATCC 25922 infection, D-xylose obviously increased DNA copies of phages Φ1, Φ2, Φ3, and total phages by up to approximately 10.87-fold (3.33 × 10^6^_Con_ versus 3.62 × 10^7^_Xyl_ copies/100 mg feces), 17.7-fold (1.81 × 10^8^_Con_ versus 3.21 × 10^9^_Xyl_ copies/100 mg feces), 9.2-fold (3.0 × 10^9^_Con_ versus 2.76 × 10^10^_Xyl_ copies/100 mg feces), and 11-fold (3.59 × 10^9^_Con_ versus 3.98 × 10^10^_Xyl_ copies/100 mg feces), respectively in mice (*P* < 0.001) (Supplementary Fig. 8), and increased phage Φ1, Φ2, Φ3, and total phage:*E. coli* DNA ratios (*P* < 0.001) (Fig. 1o–r). Importantly, we did not observe any gene amplification of phages Φ1, Φ2, and Φ3 in the PBS and D-xylose control groups, suggesting that detected fecal phage DNA was derived from released phage particles from *E. coli* ATCC 25922 (Supplementary Fig. 8). Collectively, these results demonstrated that D-xylose promotes phage production in *E. coli* ATCC 25922 and reduces *E. coli* ATCC 25922 survival in the gut.

### D-Xylose-mediated prophage induction is in D-lactic acid-dependent manner

**Fig. 2:**
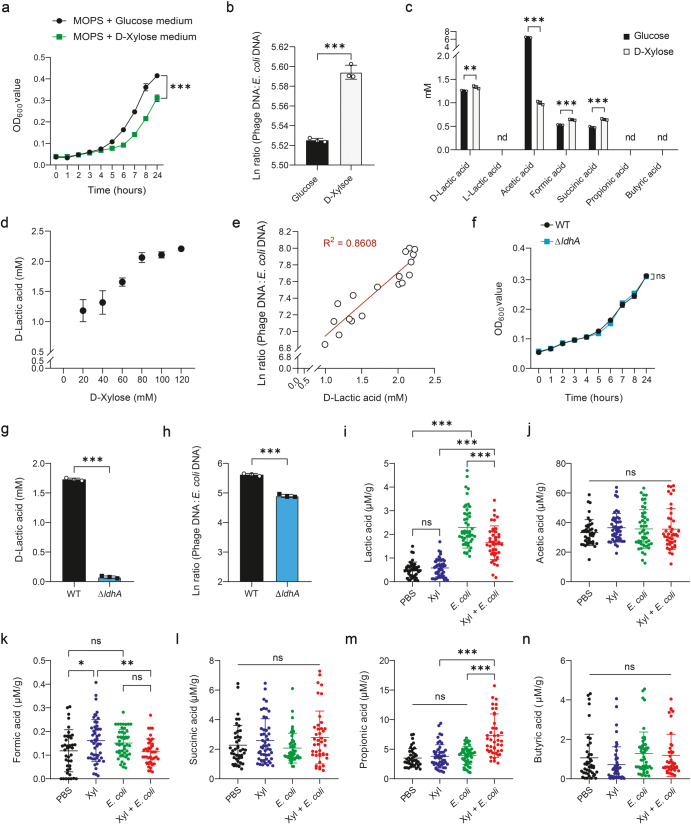
D-Lactic acid is associated with prophage induction. **a** Growth curves of *E. coli* ATCC 25922 in MOPS minimal medium supplemented with 20 mM D-xylose or glucose (*n* = 3). Total phage (sum of phage Φ1, Φ2, and Φ3):*E. coli* DNA ratio (/mL) (**b**) and metabolic end products (**c**) of *E. coli* ATCC 25922 upon 24 h growth in MOPS minimal medium containing 20 mM D-xylose or glucose (*n* = 3, respectively). **d** D-Lactic acid production of *E. coli* ATCC 25922 upon 24 h growth in MOPS minimal medium containing 0, 20, 40, 60, 80, 100, and 120 mM D-xylose (*n* = 3). **e** Correlation analysis between D-lactic acid and total phage:*E. coli* DNA ratio (/mL) (*n* = 3). **f** Growth of wild-type (WT) and *ldhA*-deficient (Δ*ldhA*) strains of *E. coli* ATCC 25922 in MOPS minimal medium containing 100 mM D-xylose (*n* = 3). D-Lactic acid production (**g**) and total phage:*E. coli* DNA ratio (/mL) (**h**) of wild-type and *ldhA*-deficient strains of *E. coli* ATCC 25922 upon 24 h growth in MOPS minimal medium supplemented with 100 mM D-xylose (*n* = 3, respectively). **i**–**n** Characterization of fecal metabolites in mice during an experimental period of 7 days. Six/seven mice were used for each treatment group. See “Methods” section for more details. Each dot in (**i**–**n**) represents a single data point from a single mouse fecal sample on one day. nd not detectable. Data in (**a**–**c**, **f**–**n**) were analyzed using unpaired Studentʼs *t* test. Data in (**e**) were analyzed using linear regression analysis. ns not statistically significant. *P < 0.05, **P < 0.01, ***P < 0.001. Data were expressed as mean ± SD.

To better understand D-xylose metabolism in bacteria, *E. coli* ATCC 25922 was cultured in MOPS minimal medium supplemented with D-xylose as the sole carbon source. We observed that *E. coli* ATCC 25922 could metabolize D-xylose, evidenced by decreased D-xylose concentration (Supplementary Fig. 9). However, D-xylose impaired *E. coli* growth and increased phage:*E. coli* DNA ratio compared with glucose (Fig. 2a, b). To understand how D-xylose affects prophage induction in *E. coli* ATCC 25922, metabolic end products in culture medium were analyzed. Results showed that D-xylose treatment significantly increased D-lactic acid, formic acid, and succinic acid production (*P* < 0.01), with D-lactic acid as the most abundant end product (Fig. 2c and Supplementary Fig. 19a). Furthermore, supplementation with increasing concentrations of D-xylose gradually increased D-lactic acid production (Fig. 2d). Phage:*E. coli* DNA ratio was significantly positively correlated with D-lactic acid level (*R*^2^ = 0.8608) (*P* < 0.001) (Fig. 2e), indicating that D-lactic acid may be responsible for regulating prophage induction.

Next, we inactivated D-lactate dehydrogenase (Δ*ldhA* mutant) to investigate whether D-lactic acid production by *E. coli* ATCC 25922 triggers prophage induction. The Δ*ldh*A mutant showed no observable growth defects compared with the wild-type strain (Fig. 2f). Deletion of *ldh*A gene almost completely inhibited D-lactic acid production (*P* < 0.001) (Fig. 2g) and highly significantly reduced phage:*E. coli* DNA ratio (*P* < 0.001) (Fig. 2h). With the increase of D-lactic acid addition in LB medium, cell density was gradually reduced (Supplementary Fig. 10a) and phage:*E. coli* DNA ratio was increased (Supplementary Fig. 10b). These data suggest that D-xylose-mediated prophage induction in *E. coli* ATCC 25922 is partially dependent upon D-lactic acid. However, dietary D-xylose reduced fecal lactic acid levels in mice infected with *E. coli* ATCC 25922 (*P* < 0.001) (Fig. 2i), but acetic acid, formic acid, succinic acid, and butyric acid were not affected (Fig. 2j–l, n), except for propionic acid, which was significantly increased in *E. coli*-challenged mice in response to D-xylose supplementation (*P* < 0.001) (Fig. 2m). Thus, D-xylose drives different metabolic profiles of lactic acid and propionic acid between in vitro and in vivo.

### D-Lactic acid generated from D-xylose metabolism by *E. coli* ATCC 25922 provides the substrate for propionic acid synthesis

**Fig. 3:**
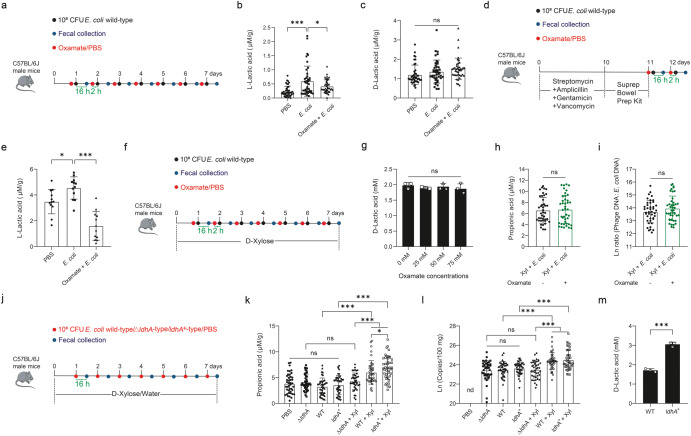
*E. coli* ATCC 25922-metabolized D-lactic acid is a substrate required for propionic acid synthesis. **a** Experimental timeline examining effects of *E. coli* ATCC 25922 challenge on D-lactic acid and L-lactic acid production in the wild-type mice. Six mice were used for each treatment group. Fecal L-lactic acid (**b**) and D-lactic acid production (**c**) during an experimental period of 7 days. **d** Experimental timeline examining the effect of *E. coli* ATCC 25922 challenge on L-lactic acid production in the gut microbiota-depleted mice. Six mice were used for each treatment group. **e** Fecal L-lactic acid production. **f** Experimental timeline examining effects of sodium oxamate on propionic acid production and prophage induction. Mice were intragastrically administered daily with either 100 µL (12 mg) sodium oxamate or phosphate buffer saline 2 h prior to *E. coli* challenge. Six mice were used for each treatment group. **g** D-Lactic acid production of *E. coli* ATCC 25922 upon 24 h growth in MOPS + D-xylose (Xyl) (100 mM) medium containing 0, 25, 50, and 75 mM sodium oxamate. Fecal propionic acid concentration (**h**) and total phage (sum of phage Φ1, Φ2, and Φ3):*E. coli* DNA ratio (/100 mg) (**i**) during an experimental period of 7 days. **j** Experimental timeline examining effects of the *ldhA* gene of *E. coli* ATCC 25922 on propionic acid production and phage production in response to D-xylose. Mice were administered with 10^8^ CFU of wild-type (WT), *ldhA*-deficient (Δ*ldhA*), or *ldhA*-overexpressing (*ldhA*^+^) strain of *E. coli* ATCC 25922 daily for 7 consecutive days while having free access to D-xylose in drinking water. Six mice were used for each treatment group. Fecal propionic acid concentration (**k**) and total phage production (**l**) during an experimental period of 7 days. **m** D-Lactic acid production of *E. coli* ATCC 25922 wild-type strain and *ldhA*-overexpressing strain upon 24 h growth in MOPS minimal medium containing 100 mM D-xylose (*n* = 3). Each dot in (**b**, **c**, **e**, **h**, **i**, **k**, **l**) represents a single data point from a single mouse fecal sample on one day. nd not detectable. Data in (**b**, **c**, **e**, **h**, **i**, **k**–**m**) were analyzed using unpaired Studentʼs *t* test. Data in (**g**) were analyzed using one-way ANOVA with Tukey’s test. ns not statistically significant. *P < 0.05, **P < 0.01, ***P < 0.001. Data were expressed as mean ± SD.

Lactic acid is one of a substrate for propionic acid synthesis and can be generated by both bacterial and mammalian cells^26^. We therefore investigated the source of lactic acid that was possibly converted to propionic acid and further explored whether prophage induction would change with varied intestinal propionic acid levels. First, dietary D-xylose had no effect on the synthesis of lactic acid or propionic acid (Fig. 2i, m and Supplementary Fig. 11) in healthy mice, suggesting that the gut microbiota does not utilize D-xylose directly to synthesize lactate or propionate. To assess whether host-derived L-lactic acid is used to synthesize propionic acid in *E. coli*-infected mice, we first treated mice with oxamate to block L-lactic acid production by inhibiting L-lactate dehydrogenase, followed by *E. coli* ATCC 25922 infection (Fig. 3a). Infection caused a significant increase in L-lactic acid (*P* < 0.001), but not bacterial-derived D-lactic acid, while oxamate largely abolished *E. coli*-induced synthesis of L-lactic acid with no impact on D-lactic acid (Fig. 3b, c).

To directly confirm whether the gut microbiota is involved in *E. coli*-induced L-lactic acid synthesis, we depleted the gut microbiota first with a cocktail of antibiotics, followed by administration of oxamate and *E. coli* ATCC 25922 (Fig. 3d). Surprisingly, *E. coli* infection still significantly increased L-lactic acid synthesis in the microbiota-depleted mice (*P* < 0.05) (Fig. 3e), suggesting that the host, rather than the gut microbiota, is likely to be responsible for increased L-lactic acid production in *E. coli*-infected mice.

To further examine whether host-derived L-lactic acid is involved in D-xylose-enhanced propionate synthesis in *E. coli*-infected mice, we tested the effect of oxamate on D-lactic acid production of *E. coli* in vitro and subsequently supplemented mice with D-xylose, followed by oxamate and *E. coli* treatments (Fig. 3f). Results showed that oxamate had no impact either on the concentrations of D-lactic acid when *E. coli* ATCC 25922 metabolized D-xylose in MOPS medium (Fig. 3g), or fecal propionic acid (Fig. 3h) and phage:*E. coli* DNA ratio (Fig. 3i) in *E. coli*-infected mice supplemented with D-xylose, suggesting that host-derived L-lactic acid is not the substrate for the synthesis of propionic acid or induction of phages in *E. coli*-infected mice.

To evaluate whether commensal bacteria utilizes D-lactic acid produced by *E. coli* ATCC 25922 to produce propionic acid, we challenged mice with wild-type, *ldhA*-deficient (Δ*ldhA*), or *ldhA*-overexpressing (*ldhA*^+^) mutant strains of *E. coli* ATCC 25922 with or without a supplementation of D-xylose in drinking water (Fig. 3j). We found that the fecal propionic acid was unaffected by infection with any *E. coli* ATCC 25922 strains when mice were not supplemented with D-xylose (Fig. 3k). However, the enzymatic activity of *ldhA* was positively associated with propionate production among infected mice supplemented with D-xylose (Fig. 3k). The fecal propionate level was the highest in mice challenged with *ldhA*^+^ mutant, but significantly diminished in Δ*ldhA*-challenged mice to the levels that were comparable to those mice with no D-xylose supplementation (Fig. 3k). Phage production was also altered among different groups in an almost identical trend to propionic acid (Fig. 3l). It is noted that *ldhA*^+^ mutant of *E. coli* ATCC 25922 indeed promoted gene expression of *ldhA* (*P* < 0.001) (Supplementary Fig. 12) and D-lactic acid production in MOPS medium containing D-xylose (*P* < 0.001) (Fig. 3m). Taken together, these results suggested that *E. coli* ATCC 25922 metabolizes D-xylose to produce D-lactic acid, which is in turn utilized by the gut microbiota to generate propionic acid.

### Clostridia converts D-lactic acid to propionic acid

**Fig. 4:**
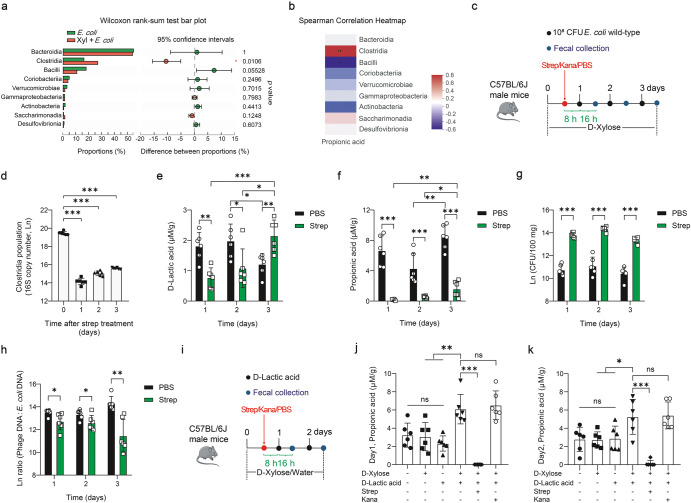
Depletion of Clostridia reduces intestinal propionic acid levels and leads to the accumulation of D-lactic acid. **a** Wilcoxon rank-sum test bar plot at the class level. **b** Correlation analysis between propionic acid production and fecal microbiota composition in D-xylose (Xyl) + *E. coli* treatment group. **c** Experimental timeline examining effects of streptomycin (Strep) or kanamycin (Kana) treatment on intestinal D-lactic acid and propionic acid concentrations. Six mice were used for each treatment group. See “Methods” for more details. **d** RT-qPCR analysis of fecal Clostridia copies after streptomycin treatment. Fecal D-lactic acid concentration (**e**), propionic acid concentration (**f**), *E. coli* ATCC 25922 number (**g**), and total phage (sum of phage Φ1, Φ2, and Φ3): *E. coli* DNA ratio (/100 mg) (**h**). **i** Experimental timeline examining the effect of oral administration of D-lactic acid on intestinal propionic acid concentration. Six mice were used for each treatment group. Fecal propionic acid concentration on day 1 (**j**) and day 2 (**k**). Data in (**a**) represent the mean values of 7 fecal samples. Each dot in (**d**–**h**, **j**, **k**) represents a single data point from a single mouse fecal sample. Data in (**b**) were analyzed using Spearman correlation analysis. Data in (**d**) were analyzed using one-way ANOVA with Tukey’s test. Data in (**e**–**h**, **j**, **k**) were analyzed using unpaired Studentʼs *t* test. ns not statistically significant. *P < 0.05, **P < 0.01, ***P < 0.001. Data were expressed as mean ± SD.

To further investigate which gut bacteria are responsible for the conversion of D-lactic acid to propionic acid, we assayed fecal microbial composition between *E. coli* and D-xylose + *E. coli* treatments. Results from the Wilcoxon rank-sum test analysis and the linear discriminant analysis effect size (LEfSe) analysis showed that D-xylose treatment only markedly increased the proportion of class Clostridia (*P* < 0.05), which was increased by more than 66.75% (Fig. 4a and Supplementary Fig. 13). Importantly, Clostridia had a significantly positive correlation with propionic acid production (*P* < 0.01) (Fig. 4b). At the genus level, increased bacteria belonging to Clostridia was *unclassified_f_Lachnospiraceae* (*P* < 0.05) (Supplementary Fig. 14). Thus, we hypothesized that ablation of Clostridia might reduce the accumulation of propionic acid. To test this hypothesis, wild-type mice were supplemented with D-xylose and then administered with streptomycin to remove Gram-positive Clostridia^27^, followed by *E. coli* infection (Fig. 4c). Fecal Clostridia was significantly reduced for three consecutive days following a single streptomycin treatment (*P* < 0.001) (Fig. 4d). Concomitant with the depletion of Clostridia, the fecal level of D-lactic acid was reduced, but gradually restored in the streptomycin group and exceeded that in the PBS group on day 3 (*P* < 0.01) (Fig. 4e). Streptomycin treatment almost completely eliminated the production of propionic acid (*P* < 0.001) (Fig. 4f). Compared to day 2, PBS treatment decreased D-lactic acid production (*P* < 0.05) and increased propionic acid production (*P* < 0.01) on day 3 (Fig. 4e, f). Both the number of *E. coli* ATCC 25922 and phage production were significantly increased (*P* < 0.01) (Fig. 4g and Supplementary Fig. 15). However, phage:*E. coli* DNA ratio was significantly reduced after streptomycin treatment (*P* < 0.05) (Fig. 4h). In contrast, oral kanamycin that mainly targets Gram-negative bacteria had no or limited impact on the production of D-lactic acid and propionic acid (Supplementary Fig. 16), both of which showed a highly similar variation trend in comparison to the PBS group over time (Supplementary Fig. 16b, c). Therefore, depletion of Clostridia correlates with reduced intestinal propionic acid levels and leads to the accumulation of D-lactic acid.

To directly confirm the role of Clostridia in the conversion of D-lactic acid to propionic acid, we gavaged wild-type, streptomycin or kanamycin-treated mice with sodium D-lactic acid (10 mg/day) and added D-xylose to their drinking water (Fig. 4i). Results showed that oral administration of sodium D-lactic acid increased fecal propionic acid production either in wild-type mice or in kanamycin-treated mice with D-xylose supplementation, but had no effect on propionic acid production in wild-type mice without D-xylose supplementation (*P* > 0.05) (Fig. 4j, k and Supplementary Fig. 17). The fecal propionic acid levels were largely reduced after streptomycin, sodium D-lactic acid, and D-xylose treatments (*P* < 0.001) (Fig. 4j, k). Furthermore, D-xylose significantly promoted propionic acid production in *E. coli* ATCC 25922-Clostridium symbiosum co-culture system (*P* < 0.001) (Supplementary Fig. 18). Overall, these experiments support the idea that Clostridia converts D-lactic acid to propionic acid **(**Supplementary Fig. 19b).

### Propionic acid promotes prophage induction in *E. coli* ATCC 25922

**Fig. 5:**
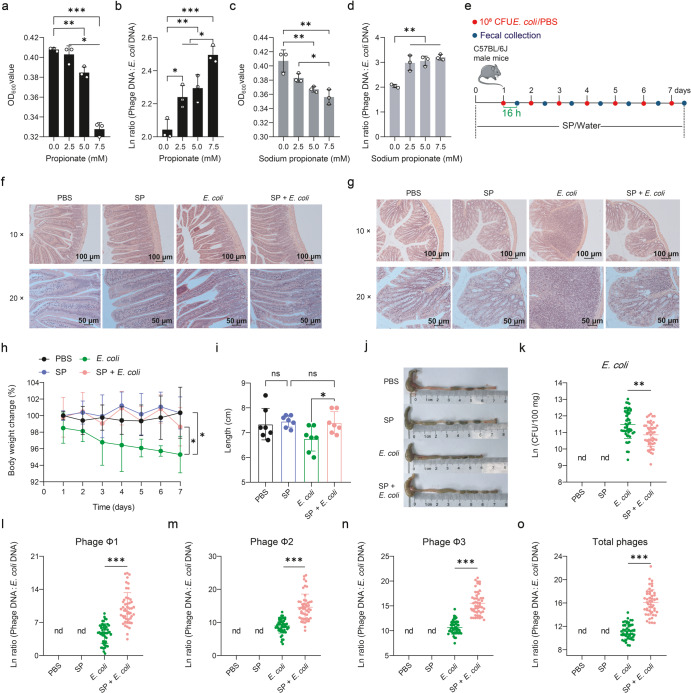
Propionic acid promotes prophage induction in *E. coli* ATCC 25922. Cell density of *E. coli* ATCC 25922 (**a**) and total phage (sum of phage Φ1, Φ2, and Φ3):*E. coli* DNA ratio (/mL) (**b**) upon 24 h growth in Lysogeny Broth (LB) medium supplemented with 0, 2.5, 5.0, and 7.5 mM propionic acid (*n* = 3, respectively). Cell density of *E. coli* ATCC 25922 (**c**) and total phage:*E. coli* DNA ratio (/mL) (**d**) upon 24 h growth in LB medium supplemented with 0, 2.5, 5.0, and 7.5 mM sodium propionate (*n* = 3, respectively). **e** Experimental timeline examining the effect of sodium propionate (SP) on prophage induction in *E. coli* ATCC 25922. Seven mice were used for each treatment group. See “Methods” for more details. Representative H&E staining images (Scale bars = 100 μm) of jejunum (**f**) and colon (**g**) and corresponding local high magnification images (Scale bars = 50 μm), body weight change (**h**), colon length (**i**), and representative colon images (**j**). Number of *E. coli* ATCC 25922 (**k**) and phage Φ1, Φ2, Φ3, and total phage:*E. coli* DNA ratios (/100 mg) (**l**–**o**) in feces during an experimental period of 7 days. Each dot in (**h**) represents the mean value of 7 mice per group daily. Each dot in (**i**) represents a single data point from a single mouse. Each dot in (**k**–**o**) represents a single data point from a single mouse fecal sample on one day. nd not detectable. Data in (**a**–**d**) were analyzed using one-way ANOVA with Tukey’s test. Data in (**h**, **i**, **k**–**o**) were analyzed using unpaired Studentʼs *t* test. ns not statistically significant. *P < 0.05, **P < 0.01, ***P < 0.001. Data were expressed as mean ± SD.

To further test whether propionic acid can induce phage production in *E. coli* ATCC 25922, we supplemented LB medium with propionic acid or sodium propionate (pH 6.95) at different concentrations (0, 2.5, 5.0, and 7.5 mM) for 24 h. Albeit with suppression of bacterial growth (Fig. 5a, c), both propionic acid and sodium propionate promoted phage production (Supplementary Fig. 20) and increased phage:*E. coli* DNA ratio (Fig. 5b, d). These data suggest that propionic acid triggers prophage induction in *E. coli* ATCC 25922, independent or non-independent of pH. To directly test whether dietary propionate can enhance phage production in live animals, we supplemented sodium propionate to mice in their drinking water (20 mg/mL)^28^, followed by challenge with *E. coli* ATCC 25922 (Fig. 5e). We found that sodium propionate significantly reversed body weight loss (Fig. 5h), reduced the levels of serum inflammatory factors (i.e., TNF-α, IL-6, IL-10, and IL-1β) (Supplementary Fig. 21a–d), suppressed liver enlargement (Supplementary Fig. 21e, f), and inhibited colon shortening (Fig. 5i, j) as well as intestinal pathological changes induced by *E. coli* ATCC 25922 treatment (Fig. 5f, g). Importantly, fecal *E. coli* ATCC 25922 counts were significantly decreased (*P* < 0.01) (Fig. 5k and Supplementary Fig. 5b), and the copies of phage Φ1, Φ2, Φ3, and total phages (*P* < 0.001) (Supplementary Fig 22) as well as phage Φ1, Φ2, Φ3, and total phage:*E. coli* DNA ratios (*P* < 0.001) (Fig. 5l–o) were drastically increased, suggesting that propionic acid promotes prophage induction in *E. coli* ATCC 25922.

### The SOS response is involved in prophage induction in *E. coli* ATCC 25922

**Fig. 6:**
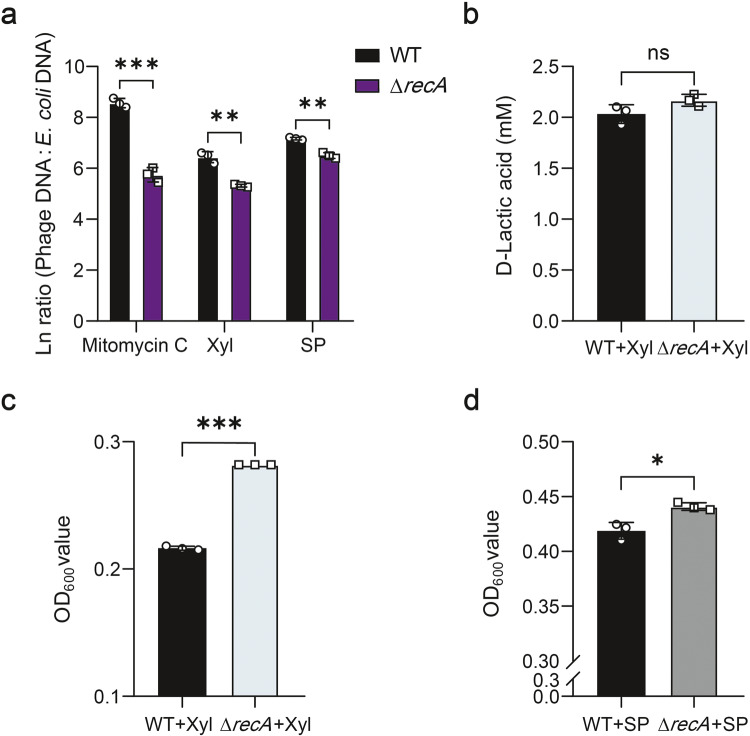
Prophage induction in *E. coli* ATCC 25922 is in RecA-dependent manner. **a** Total phage (sum of phage Φ1, Φ2, and Φ3):*E. coli* DNA ratio (/mL) in culture medium supplemented with mitomycin C (0.5 µg/mL), 100 mM D-xylose (Xyl), and 7.5 mM sodium propionate (SP) (*n* = 3). D-Lactic acid production (**b**) and cell density (**c**) of *E. coli* ATCC 25922 wild-type (WT) and Δ*recA*-type upon 24 h growth in MOPS minimal medium supplemented with 100 mM D-xylose (*n* = 3, respectively). **d** Cell density of *E. coli* ATCC 25922 wild-type and Δ*recA*-type upon 24 h growth in LB medium supplemented with 7.5 mM sodium propionate (*n* = 3). All data were analyzed using unpaired Studentʼs *t* test. ns not statistically significant. *P < 0.05, **P < 0.01, ***P < 0.001. Data were expressed as mean ± SD.

Many prophages maintain a stable state benefiting from the silence of the SOS response^15,29^. Activation of the SOS system is commonly associated with phage production^30^. In *E. coli*, the key regulatory protein of the SOS system is RecA^31^. To determine whether prophage induction in *E. coli* ATCC 25922 is dependent upon RecA, we generated a *recA*-deficient (Δ*recA*) mutant. The Δ*recA* mutant showed markedly reduced phage production (*P* < 0.05) (Supplementary Fig. 23) and phage:*E. coli* DNA ratios (*P* < 0.01) (Fig. 6a) following mitomycin C, D-xylose, or sodium propionate treatments compared with the wild-type strain. However, D-lactic acid production from D-xylose was not affected in the Δ*recA* mutant (*P* > 0.05) (Fig. 6b). Interestingly, the Δ*recA* mutant grew even better than that of the wild-type strain in MOPS medium containing D-xylose as the only carbon source (*P* < 0.001) (Fig. 6c) or in LB medium supplemented with sodium propionate (*P* < 0.05) (Fig. 6d). These findings indicate that the SOS system is involved in the induction of prophages of *E. coli* ATCC 25922 in response to dietary D-xylose and intestinal propionic acid.

### Inhibition of *recA*-dependent prophage activation aggravates *E. coli* ATCC 25922-induced systematic inflammation

**Fig. 7:**
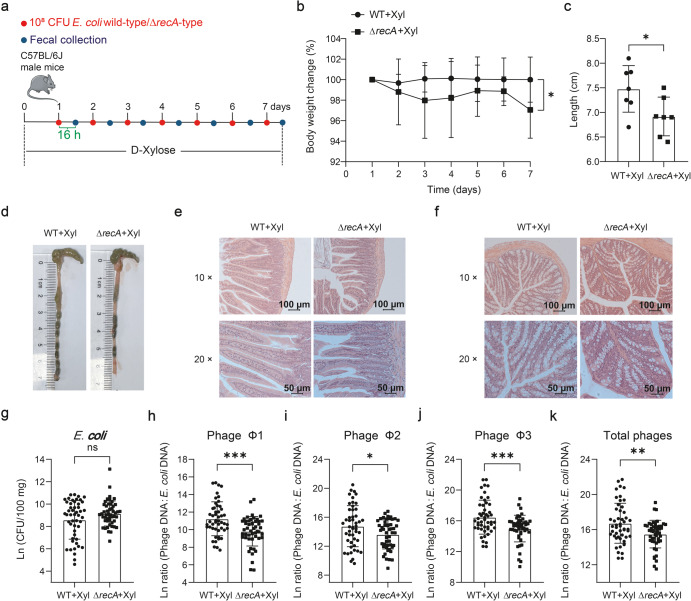
Suppression of prophage induction in *E. coli* ATCC 25922 deteriorates intestinal inflammation response. **a** Experimental timeline examining the effect of *recA*-knockout on *E. coli* ATCC 25922-mediated intestinal inflammation response. Seven mice were used for each treatment group. Body weight change (**b**), colon length (**c**), representative colon images (**d**), and representative H&E staining images (Scale bars = 100 μm) of jejunum (**e**) and colon (**f**) and corresponding local high magnification images (scale bars = 50 μm). **g** *E. coli* ATCC 25922 number in feces during an experimental period of 7 days. Phage Φ1, Φ2, Φ3, and total phage (sum of phage Φ1, Φ2, and Φ3):*E. coli* DNA ratios (/100 mg) (**h**–**k**) in feces during an experimental period of 7 days. Each dot in (**b**) represents the mean value of 7 mice per group daily. Each dot in (**c**) represents a single data point from a single mouse. Each dot in (**g**–**k**) represents a single data point from a single mouse fecal sample on one day. All data were analyzed using unpaired Studentʼs *t* test. ns not statistically significant. *P < 0.05, **P < 0.01, ***P < 0.001. Data were expressed as mean ± SD.

Since D-xylose and propionic acid were capable of promoting phage production in *E. coli* ATCC 25922 and inhibiting bacterial growth, we hypothesized that suppressing prophage activation might enhance bacterial survival and exacerbate systematic inflammation. To test this hypothesis, we first evaluated whether *recA* affects the pathogenicity of *E. coli* ATCC 25922 and found that it had a negligible impact on the pathology in mice (Supplementary Fig. 24). To further explore *recA*-induced changes in phage production and *E. coli* counts, mice were gavaged with the wild-type or Δ*recA* mutant strain while having ad libitum access to D-xylose in drinking water (Fig. 7a). We observed more severe inflammatory symptoms (Fig. 7b–f), higher levels of fecal *E. coli* ATCC 25922 (*P* > 0.05) (Fig. 7g), and lower phages (*P* < 0.05) (Supplementary Fig. 25) as well as phage:*E. coli* DNA ratios (*P* < 0.05) (Fig. 7h–k) in mice that was received the Δ*recA* mutant. Overall, these data demonstrated that prophage induction in *E. coli* ATCC 25922 contributes to D-xylose-mediated antimicrobial effects.

### D-Xylose-mediated prophage induction in *E. coli* is strain-specific

To investigate whether D-xylose is specific to *E. coli* ATCC 25922, we also tested enteropathogenic *E. coli* (EPEC) LHM10-1 which encodes three complete prophages and enteroinvasive *E. coli* (EIEC) CP66-6 which is lysogenized with two complete prophages. Supplementation with D-xylose in LB medium only reduced *E. coli* number (Supplementary Fig. 26a) and increased phage production (Supplementary Fig. 26b) as well as phage:*E. coli* DNA ratio (Supplementary Fig. 26c) in *E. coli* LHM10-1, but not in *E. coli* CP66-6 (Supplementary Fig. 26d–f), suggesting that D-xylose-mediated prophage induction in *E. coli* is strain-specific.

## Discussion

In recent years, several studies have indicated that prophage induction plays important roles in regulating the abundance or diversity of intestinal bacteria^15,32^. On the one hand, prophage induction is one of the important causes of gut microbiota dysbiosis. For example, in inflammatory bowel disease patients, prophage induction was tightly related to depletion of *Faecalibacterium prausnitzii*, one of the major commensal bacteria in human gut^32^. During gastrointestinal transit, phage production reduced the survival of *Lactobacillus reuteri*^15^. On the other hand, prophage induction may impair survival advantages of the pathogen. A previous study has shown that Stevia induced the formation of virus-like particles in *Staphylococcus aureus* and inhibited bacterial growth in Brain–Heart Infusion Broth^14^. Therefore, target modulation of prophage induction appears crucial to gut microbiome homeostasis and intestinal health. Furthermore, in a large-scale in vitro screening test, many commonly consumed foods or compounds were found to be prophage inducers or inhibitors, implying the feasibility of using dietary components to intentionally manipulate prophage induction^14^. D-Xylose has been used to treat metabolic syndrome and diabetes^17,33^. However, few studies have examined the effects of dietary D-xylose supplementation on intestinal microbial composition and alterations in the phageome. Our work provides evidence that dietary D-xylose reduced the survival of *E. coli* ATCC 25922 by facilitating prophage induction, thereby promoting intestinal health.

We shed light on several potential causes of D-xylose-mediated prophage induction in *E. coli* ATCC 25922. In vitro, *E. coli* utilized D-xylose to produce D-lactic acid which promoted prophage induction. However, a notable level of phages could be produced by *E. coli* ATCC 25922 growth in MOPS minimal medium without D-xylose supplementation. The MOPS minimal medium lacks some nutrients, for example, amino acids et al. Previous study has shown that deprivation of specific nutrients such as lysine, leucine, methionine, proline, or tryptophan effectively promoted lambda prophage induction^34^. Therefore, the lack of specific nutrients in MOPS minimal medium is a potential cause for prophage induction in *E. coli* ATCC 25922 even though no D-xylose was added. Dietary D-xylose induced the production of propionic acid in the gut, which further strengthened prophage induction in *E. coli* ATCC 25922. This may explain why the increased magnitude of phage production in vivo (11-fold) was much higher than that observed in vitro (2.13-fold). Intriguingly, microbial cross-feeding was observed between *E. coli* ATCC 25922 and Clostridia in dietary D-xylose intervention study. Intestinal Clostridia converted D-lactic acid produced from D-xylose metabolism by *E. coli* ATCC 25922 to propionic acid. Propionate can be synthesized through the acrylate pathway, in which lactic acid is converted to propionic acid^26^. The acrylate pathway has only been identified in a handful of families, including *Lachnospiraceae* and *Veillonellaceae*^35^. These facts support our finding that Clostridia (*Lachnospiraceae*) converted D-lactic acid to propionic acid. Similar phenomenon was previously reported, where dietary supplementation with xylitol enhanced propionic acid production in the murine colon through cross-feeding from microbiota^36^. Noteworthy, despite propionic acid-mediated prophage activation being eliminated by streptomycin treatment, both *E. coli* ATCC 25922 number and phage production were extremely increased. The magnitude of the increase in the number of *E. coli* ATCC 25922 outweighed its decrease after propionic acid treatment. This suggests that apart from prophage induction, gut commensal microbe might have multiple mechanisms to resist the colonization of *E. coli* ATCC 25922. Increased phage production is therefore likely attributable to D-xylose metabolism or other factors-mediated prophage induction in a large population of living *E. coli* ATCC 25922. Collectively, cross-feeding interactions in microbial communities may alter gut microbiota composition by modulating prophages.

There is a growing body of evidence that the composition of gut virome can be influenced by diet^12,14,15^. However, the nutritional mechanisms that induce intestinal prophages remain largely unexplored. Our data indicated that the prophage induction in *E. coli* ATCC 25922 required for the involvement of SOS system. A recent work demonstrated that fructose-induced phage production in *Lactobacillus reuteri* was SOS response-dependent, which was in line with our observations^15^. Nonetheless, in our research, there are some evidence indicating that the mechanisms of prophage induction in *E. coli* ATCC 25922 were independent upon the SOS response. First, the knockout of *recA* partly but not completely inhibited phage production. Second, the pH changes in the intestinal environment are known to cause bacterial DNA damage and activation of the SOS response^37^. However, increased levels of L-lactic acid slightly stimulated the production of phages during *E. coli* ATCC 25922 infection. As the first responder to pathogen infection, neutrophils will undergo enhanced glycolysis^38^. *E. coli* ATCC 25922 infection-induced glycolysis of neutrophils might contribute to the accumulation of L-lactic acid. Finally, it is worth noting that the production of phages Φ2 and Φ3 were always higher than that of phage Φ1, suggesting prophage-specific activation mechanisms. Further experiments should be conducted to elucidate these points. Indeed, several mechanisms of *recA*- or SOS-independent prophage induction have been demonstrated, for example, via relieving *pir* inhibition, enhancing expression of QseE or phage-encoded Qtip, as reviewed by Henrot and Petit^39^. The SOS pathway not only participates in regulating prophage induction, but may also play critical roles in various important bacterial activities, such as DNA damage response and genetic variation^31^. Although knockout of *recA* gene may weaken the activation of prophages in *E. coli* ATCC 25922, due to the potential existence of abundant DNA damaging agents in the intestine, it brings a burden to bacterial survival. Therefore, this may lead to a less pronounced growth advantage of *E. coli* ATCC 25922 Δ*recA* mutant in response to dietary D-xylose and intestinal propionic acid than in vitro experiments.

In some disease states, microorganisms that are detrimental to intestinal health may even include normal gut microbiota. For example, the competition between *Vibrio cholerae* and host commensal bacteria promoted the pathogenesis of *Vibrio cholera*^40^. In turn, the removal of commensal bacteria attenuated disease severity. Another interesting example is that gut symbiotic bacteria *Bacteroides fragilis* was selectively enriched at an early stage in patients suffering from colorectal cancer and exacerbated inflammatory response^41^. At present, we have few strategies to prevent or control bacterial functional changes. While D-xylose may be specific to induce prophages in certain pathogenic *E. coli*, screening of more dietary prophage inducers is promising to reduce undesired lysogenic gut bacteria, suggesting a potential clinical implication for treating bacteria-associated diseases. Released temperate phages may modify gut bacterial communities by multiple life cycles, for example, transduction, lysogenic conversion, and lytic life cycles^16^. Therefore, understanding the roles of released phages on intestinal health is also critical. Further research is required to elucidate this.

In this research, we explained the underlying mechanisms by which D-xylose induced phage production in *E. coli* ATCC 25922. D-Xylose metabolism by *E. coli* ATCC 25922-mediated prophage activation was D-lactic acid-dependent. Intestinal Clostridia subsequently converted D-lactic acid to propionic acid, which in turn further activated prophages in *E. coli* ATCC 25922. Dietary D-xylose or exposure to propionic acid triggered phage production via induction of the SOS response. Our findings reveal a molecular framework for improving animal and human intestinal health through regulation of temperate phages. This study strengthens that the use of dietary prophage inducers, for example, D-xylose, can be developed as an improved therapeutic strategy for treating pathogen infection or landscaping gut microbiome.

## Methods

### Bacterial strains and growth conditions

*E. coli* ATCC 25922 (CP009072.1) was purchased from Beijing Zhongke Quality Inspection Biotechnology Co., Ltd (Beijing, China). *E. coli* ATCC 25922 and its derivatives were grown at 37 °C in 8 mL of Lysogeny Broth (LB) supplemented with or without 1% different carbon sources, including glucose (Solarbio, Beijing, China), fructose (Sigma-Aldrich, Missouri, USA), D-xylose (Aladdin, Shanghai, China), L-arabinose (Aladdin, Shanghai, China), arabinogalactan (YuanyeBiotech, Shanghai, China), and xylan (Aladdin, Shanghai, China), or 8 mL of MOPS minimal medium (Coolaber, Beijing, China) supplemented with or without D-xylose. Following 24 h incubation, bacterial samples were cooled down in an ice bath and then collected for analysis. Two hundred microliters of bacterial cultures were used to detect OD_600_ value by a microplate reader (Synergy, BioTek, USA). *E. coli* O157:H7 was obtained from a microbiology laboratory at China Agricultural University. *E. coli* LHM10-1 (CP037903.1), *E. coli* CP66-6 (CP053723.1), and Clostridium symbiosum (BNCC364164) were purchased from Beina Biotechnology (Beijing, China).

### Identification of prophages in *E. coli* ATCC 25922

Prophages in the *E. coli* ATCC 25922 genome were identified using PHAge Search Tool Enhanced Release (PHASTER, http://phaster.ca/) by entering the GenBank accession number of *E. coli* ATCC 25922 (NZ_CP009072.1) under default conditions^42^.

### Mitomycin C treatment

One single colony of *E. coli* ATCC 25922 on an LB-agar plate was cultured in 20 mL LB medium for 8 h. Bacteria was then diluted 1: 50 in fresh 100 mL LB medium and allowed to grow at 37 °C and 200 rpm till OD_600_ value reached 0.2–0.3. Mitomycin C (MedChemExpress, NJ, USA) was added to a final concentration of 0.5 µg/mL, and an aliquot (1 mL) of bacterial culture was taken at various time points, followed by centrifugation for 10 min at 1500 × *g*. The bacterial pellet was resuspended in 1 mL phosphate-buffered saline (PBS) and OD_600_ value was measured.

### Growth kinetics of *E. coli* ATCC 25922 in MOPS minimal medium supplemented with D-xylose or glucose

A colony of *E. coli* was inoculated into 8 mL LB medium and incubated for 9 h at 37 °C with shaking at 200 rpm. After centrifugation for 10 min at 1500 × *g*, 10^7^ CFU bacterial pellet was resuspended and diluted 1:100 in 8 mL MOPS minimal medium (composition available at https://www.genome.wisc.edu/resources/protocols/mopsminimal.htm) supplemented with 20 mM D-xylose or glucose and allowed to grow at 37 °C. The growth kinetics of bacteria were recorded by measuring OD_600_ value of culture medium (200 μL) at different time points.

### Co-culture experiment

*E. coli* ATCC 25922-Clostridium symbiosum co-culture experiment was performed in a 15-mL tube at 37 °C under anaerobic conditions. EG medium, composed of 0.24% (m/v) beef extract, 1% (m/v) peptone, 0.5% (m/v) yeast extract, 0.4% (m/v) Na_2_HPO_4_, 0.15% (m/v) glucose, 0.05% (m/v) soluble starch, 0.02% (m/v) *L*-cystine, 0.05% (m/v) *L*-cysteine, 5% (v/v) horse blood, or D-xylose (20 mM) was used for bacteria culture.

### Isolation and purification of phages from *E. coli* ATCC 25922 for transmission electron microscopy (TEM) detection

Bacterial cultures were centrifugated at 2350 × *g* and 4 °C for 10 min. The supernatant was filtered through a 0.22 μm membrane PVDF (Millipore), followed by 30-min incubation at room temperature (25 °C) with 1 µg/mL DNase I and 1 µg/mL RNase A to degrade bacterial DNA and RNA. The supernatant was then added with sodium chloride to a final concentration of 1 M before centrifugation at 11,000 × *g* for 10 min at 4 °C. Polyethylene glycol (PEG) 8000 (10%) was added to the supernatant and stored overnight in an ice-water bath for phage precipitation. After centrifugation at 11,000 × *g* for 10 min, phage precipitates were gently resuspended in SM buffer composed of 5.8 g/L sodium chloride, 2 g/L magnesium sulfate. 7H_2_O, 50 mL/L of 1 M Tris-Cl (pH7.5), and 0.1 g/L gelatin. An equal volume of chloroform was then added and centrifuged at 3000 × *g* for 30 s. Phage particles were harvested from the top aqueous phase and further purified by centrifugation at 110,000 × *g* and 4 °C for 2 h in Optima XPN-100 Ultracentrifuge (Beckman, Germany). Precipitated phage particles were resuspended in 1 mL of SM buffer for TEM detection.

### TEM

Negative-stain TEM was used to characterize purified phages. Specifically, 2 µL of purified phage suspension was placed on a 300 mesh carbon-coated copper grid, negatively stained for 20 s in 1% uranyl acetate, and then oven-dried at 55 °C. Electron images of phages were then recorded under an H-7650 TEM (Hitachi, Osaka, Japan).

### Dietary D-xylose and sodium propionate intervention studies

Six-week-old male C57BL/6J mice were used in this study. All animal studies were performed in accordance with the animal experimental ethics committee guidelines of China Agricultural University under protocol number AW21102202-1-1. Mice were housed under controlled conditions at 25 °C, 55 ± 5% humidity, and 12-h light/dark cycle. After a 7-d acclimation, animals were randomly divided into different groups. For dietary D-xylose intervention study: PBS (*n* = 6), D-xylose (*n* = 7), *E. coli* ATCC 25922 (*n* = 7), and *E. coli* ATCC 25922 supplemented with D-xylose (*n* = 6). For sodium propionate intervention study: PBS (*n* = 7), sodium propionate (*n* = 7), *E. coli* ATCC 25922 (*n* = 7), and *E. coli* ATCC 25922 supplemented with sodium propionate (*n* = 7). D-Xylose (5.63 mg/mL) or sodium propionate (20 mg/mL) was provided in drinking water ad libitum for 8 consecutive days (day 0 to day 7). At day 1, animals were gavaged once daily with approximately 10^8^ CFU *E. coli* ATCC 25922 for 7 days. Fecal samples were collected from each animal 16 h after bacterial gavage and resuspended in PBS to 100 mg/mL for downstream analyses. D-Xylose concentrations were determined by the d-xylose content detection kit (Enzyme-linked Biotechnology Co., Ltd, Shanghai, China). At day 7, the infected mice were anaesthetized and euthanized by cervical dislocation. Spleen, liver, blood, and intestinal tissues were collected.

### Bacteriophage DNA extraction

Phage DNA was extracted using a rapid bacteriophage genomic DNA extraction kit (Mei5 Biotechnology Co., Ltd, Beijing, China) in strict accordance with the manufacturer’s protocol. Specifically, bacterial culture and resuspended fecal samples were centrifuged at 13,500 × *g* for 10 min. The supernatant was filtered through 0.22 µm PVDF membranes (Millipore, USA). The 5 μL/mL CaCl_2_-MgCl_2_ solution (80 mM MgCl_2_ and 20 mM CaCl_2_) was added, followed by incubation with DNase I and RNase A for 30 min at 37 °C to eliminate bacterial DNA and RNA. An aliquot of the phage suspension at this stage was amplified by RT-qPCR to confirm that free bacterial DNA had been removed from the sample. Phage particles were precipitated in 10% PEG8000 and 1 M NaCl at 4 °C overnight. After centrifugation at 13,500 × *g* for 10 min at 4 °C, the pellet containing phages was lysed in the lysis buffer (4.5 M guanidinium isothiocyanate, 44 mM sodium citrate pH 7.0, 0.88% sarkosyl, 0.72% 2-mercaptoethanol), followed by incubation with 20% SDS for 10 min at 70 °C and then with ice bath for 5 min. One hundred microliters impurity precipitation solution was added to the mixture. After centrifugation at 16,000 × *g*, 4 °C for 10 min, the supernatant containing phage DNA was collected. DNA was further purified using the centrifugal adsorption column. Finally, phage DNA was eluted from the adsorption column with 48 μL preheated (50 °C) elution buffer. The concentration and purity of extracted phage DNA were determined by the NanoDrop ND-1000 spectrophotometer (NanoDrop Technologies, Wilmington, DE, USA).

### Quantification of bacteriophages by real-time quantitative PCR (RT-qPCR)

To establish a standard curve for absolute quantification of each of the three phages Φ1, Φ2, and Φ3, 173-bp, 237-bp, and 224-bp DNA fragments were amplified from each phage and cloned into pMD™19-T vector (Takara Biomedical Technology Co., Ltd, Beijing, China). Plasmids were then purified, quantified, and serially diluted in nuclease-free water to develop standard curves using RT-qPCR on a Riche light cycler 96 Real-Time PCR System (Switzerland). The 20 μL qPCR reaction mixture consisted of 0.6 µL each of the forward and reverse primers, 10 µL of SYBR-green I, 2.0 µL of DNA template (14.0–163.5 ng/μL), and 6.8 μL of nuclease-free H_2_O. The cycling program used was an initial setup of 1 min at 95 °C, followed by 40 cycles of 5 s denaturation at 95 °C, 30 s annealing at 50 °C, and 1 min extension at 72 °C. Bacteriophages were quantified under identical conditions to that of standard curves. Based on the standard curve, the Ct value was extrapolated to copy numbers of phages in each sample, given that the amplified DNA fragment of each of three phages is unique and specific. Copy numbers of phages were calculated as the numbers corresponding to that in 1 mL of original culture supernatants or 100 mg of fecal samples and shown in all relevant figures.

### Extraction of *E. coli* ATCC 25922 genomic DNA and DNA quantification

Genomic DNA of *E. coli* ATCC 25922 in liquid culture medium and fecal samples was extracted using TIANamp Bacteria DNA Kit and TIANamp Stool DNA Kit (Tiangen Biotech Co., Ltd, Beijing, China), respectively, in accordance with the instructions. The primers used for quantification of *E. coli* ATCC 25922 genomic DNA are shown in Supplementary Table 2. Quantitative experimental details were same as the bacteriophage described above.

### Analysis of metabolites

The concentrations of lactic acid, formic acid, acetic acid, propionic acid, butyric acid, and succinic acid were determined using a high-performance ion chromatography (HPIC) system (DIONEX ICS-300, Thermo Fisher, USA). The mobile phase and detector of HPIC were KOH and conductivity detector, respectively. After sonication in cold water for 30 min, the fecal mixture was centrifuged at 3000 × *g* for 10 min at 4 °C. The supernatant was diluted 1:25 with ultrapure water and filtered through a 0.22-µm membrane filter. The injection volume was 25 µL, and the flow rate was 1.0 mL/min. The bacterial culture was diluted 1:50 and filtered through a 0.22-µm membrane prior to chromatography. D-Lactic acid and L-lactic acid were analyzed using commercial kits (Enzyme-linked Biotechnology Co., Ltd, Shanghai, China).

### Measurement of serum cytokines

The serum was prepared by centrifugation of the whole blood at 2350 × *g* for 10 min. The concentrations of IL-1β, IL-6, IL-10, and TNF-α were determined using enzyme-linked immunosorbent assay (ELISA) kits (Thermo Fisher, Massachusetts, USA).

### Hematoxylin and eosin (H&E) staining

The intestinal tissues were fixed in 4% paraformaldehyde solution and then dehydrated using a graded ethanol series (70–100%). Subsequently, the samples were cleared with xylene and embedded in paraffin wax. Serial sections (5-μm thickness) were cut using LEICA RM2135 rotary microtome (Leica Microsystems GmbH), and then stained with hematoxylin and eosin. Zeiss Axio Imager microscope (Carl Zeiss Microscopy, Germany) was used to record tissue section images.

### Construction of ampicillin-resistance *E. coli* ATCC 25922 and mutant strains (Δ*ldhA*, *ldhA*^+^ and Δ*recA*)

Competent *E. coli* ATCC 25922 was prepared via the CaCl_2_ method and then transformed with pMD18-T vector (Takara Biomedical Technology Co., Ltd, Beijing, China) (https://www.genomics-online.com/vector-backbone/38/pmd18-t/) to confer ampicillin resistance. Ampicillin-resistant *E. coli* strain was used to infect mice and counted on LB-agar plates supplemented with ampicillin (100 μg/mL). Knockout of *ldhA* and *recA* genes in *E. coli* ATCC 25922 was performed using the CRISPR-B^TM^ technology (Ubigene, Guangdong, China) and homologous recombination method, respectively. Specifically, CRISPR-B^TM^ *ldhA* knockout vector was constructed first. The *ldhA*-gRNA sequence was CTGACCGGCTTTACTATGTATGG. Then, the CRISPR-B^TM^ *ldhA* knockout vector was electroporated into *E. coli* ATCC 25922 competent cells. Positive colonies were identified by colony PCR and sequencing. The plasmids pKD46, pKD3, and pCP20 used for homologous recombination were purchased from MiaoLingBio (Wuhan, China). *RecA* gene knockout primers were listed in Supplementary Table 2. To overexpress *ldhA*, *ldhA* gene was inserted into the pUC57 plasmid (Tsingke Biotechnology Co., Ltd, Beijing, China). Subsequently, the recombinant expression vector was transferred into *E. coli* ATCC 25922 cell. The plasmids were isolated with the plasmid mini kit (Omega, USA) and then analyzed by PCR and Sanger sequencing.

### 16S rRNA sequencing and analysis

Fecal content was collected and snap-frozen in liquid nitrogen. Bacterial DNA was then extracted from frozen samples using the TIANamp Stool DNA Kit (Tiangen Biotech Co., Ltd, Beijing, China). The V3–V4 region of the bacterial 16S rDNA gene was amplified with universal barcoded primers. Raw reads were subjected to quality assessment according to a standard workflow^43^. QIIME 2 (Quantitative Insights into Microbial Ecology 2) was employed to perform microbial community analysis^44^. We rarefied sequence data in QIIME 2 to a depth of 19,315 sequences per sample for 16S rRNA genes. Linear discriminant analysis (LDA) score of greater than 3.0 was used as a threshold for the linear discriminant analysis effect size (LEfSe) analysis.

### Analysis of fecal Clostridia by RT-qPCR

Fecal Clostridia was quantified using RT-qPCR and Clostridium-specific primers (sense: 5’-ACTCCTACGGGAGGCAGC-3’; antisense: 5’-GCTTCTTTAGTCAGGTACCGTCAT-3’) as described^27^. These primers specifically amplified a 140-bp DNA sequence in class Clostridia genome. Serial dilutions of the plasmids containing the 140-bp gene fragment were used to generate a standard curve for quantification of the genome copy numbers of Clostridia in fecal samples.

### Antibiotic treatment of mice

Seven-week-old male C57BL/6J mice were orally gavaged with 20 mg of kanamycin sulfate or streptomycin sulfate in 0.2 mL PBS 8 h prior to *E. coli* infection. Fresh fecal material was collected daily for analysis. To minimize the impact of antibiotics on *E. coli*, a plasmid pET28a that encodes kanamycin and streptomycin resistance genes was transformed into *E. coli* ATCC 25922.

### Antibiotic depletion of the gut microbiota

A cocktail of antibiotics, including streptomycin (1 g/L), gentamicin (1 g/L), ampicillin (0.5 g/L), and vancomycin (0.5 g/L), was provided in drinking water for 10 days to deplete the gut microbiota in mice^45^. Drinking water containing antibiotics was replaced every 2 days. To facilitate the removal of residual antibiotics on the intestinal mucosal surface, SUPREP Bowel Prep Solution comprised of sodium sulfate (262 mM), potassium sulfate (38 mM), and magnesium sulfate (28 mM) was provided in drinking water for 2 days following cessation of the antibiotic regimen before sampling. Six mice were included in each treatment group.

### Statistical analysis

Graphs were generated with GraphPad Prism 8.0 software (GraphPad Software, La Jolla, CA, USA). Statistical analyses were performed using IBM SPSS Statistics 22.0 software (Corporation, Armonk, NY). Unpaired Studentʼs *t* test was used to detect statistical significance between two treatment groups. If more than two treatment groups were involved, one-way ANOVA and post hoc Tukey’s test were performed for multiple comparisons. Data were expressed as mean ± standard deviation (SD). Values below detection limits were shown as “nd” (not detectable). Statistical significance was indicated as *P < 0.05, **P < 0.01, or ***P < 0.001. Not statistically significant was indicated as “ns”.

### Reporting summary

Further information on research design is available in the Nature Research Reporting Summary linked to this article.

### Supplementary information

Supplementary Information

Reporting Summary

## Data Availability

The dataset containing Illumina MiSeq raw reads for fecal bacterial 16S rRNA gene sequencing analyzed in this study (Fig. 4a, b and Supplementary Figs. 13a, b and 14a–c) is available in the NCBI Sequence Read Archive (SRA) under BioProject PRJNA816313. All other data that support the findings of this study are available from the corresponding author upon reasonable request.

## Code availability

No new codes were generated or used in this study.
