# Supplementary material for: Dietary D-xylose promotes intestinal health by inducing phage production in Escherichia coli
Source: NPJ Biofilms Microbiomes. 2023 Oct 11;9:79. doi: 10.1038/s41522-023-00445-w (PMC10567762; doi:10.1038/s41522-023-00445-w)
Supplement: Supplementary file 1 — Supplementary Information [file 41522_2023_445_MOESM1_ESM.pdf]

Supplementary Materials for

Dietary D-xylose promotes intestinal health by inducing phage production in *Escherichia coli*

Jie Hu, Yifan Wu, Luyuan Kang, Yisi Liu, Hao Ye, Ran Wang, Jinbiao Zhao, Guolong Zhang, Xilong Li, Junjun Wang, and Dandan Han*

*Corresponding author. Email: handandan@cau.edu.cn

This file includes:

Supplementary Figs. 1-26
Supplementary Tables 1-2

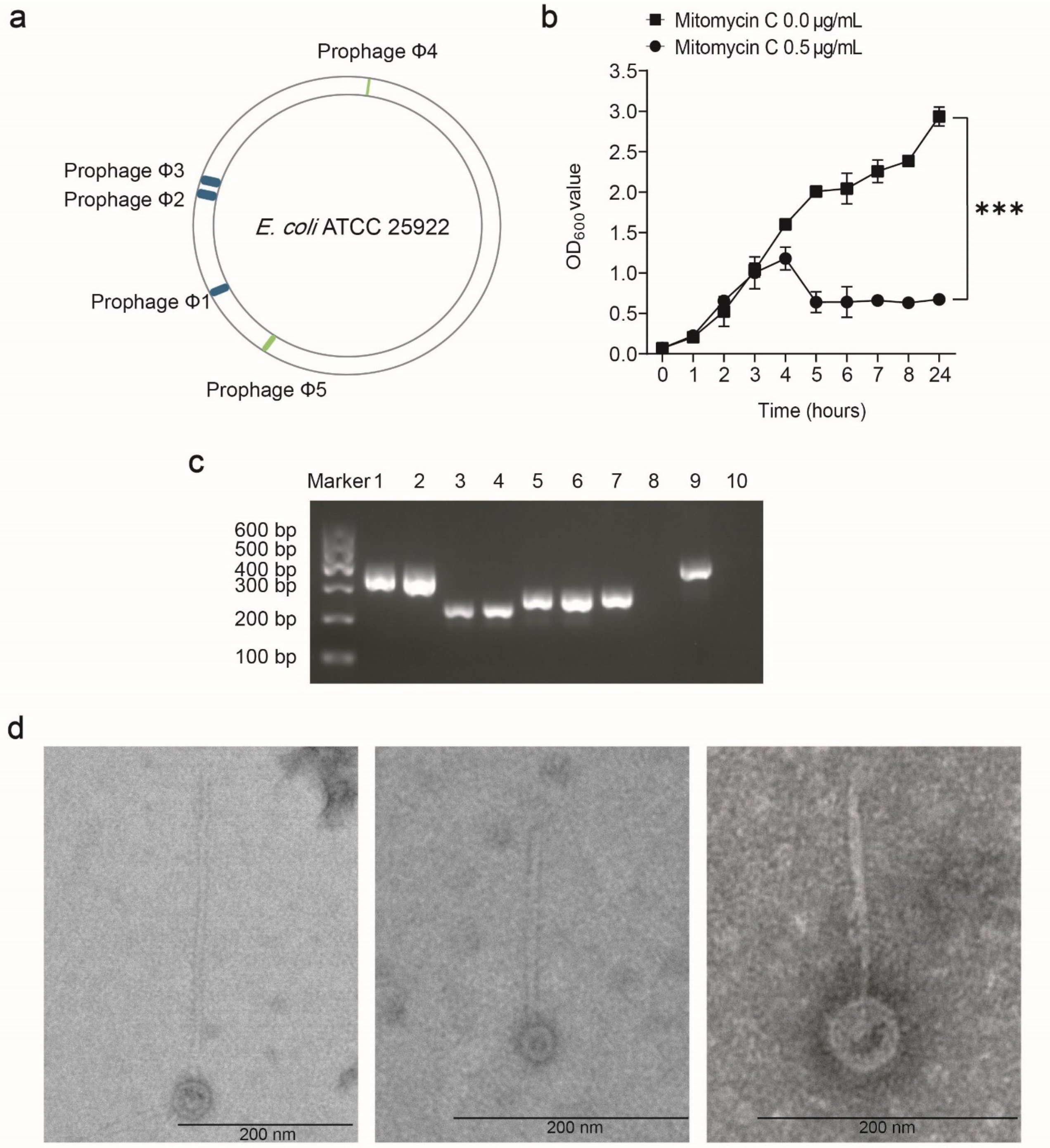
a
Prophage Φ4
Prophage Φ3
Prophage Φ2
E. coli ATCC 25922
Prophage Φ1
Prophage Φ5
b
Mitomycin C 0.0 μg/mL
Mitomycin C 0.5 μg/mL
3.5
3.0
2.5
2.0
1.5
1.0
0.5
0.0
OD600 value
***
0 1 2 3 4 5 6 7 8 24
Time (hours)
c
Marker 1 2 3 4 5 6 7 8 9 10
600 bp
500 bp
400 bp
300 bp
200 bp
100 bp
d
200 nm
200 nm
200 nm

Supplementary Fig. 1 Prophage identification in *E. coli* ATCC 25922 genome

(**a**) Prophage distribution in *E. coli* ATCC 25922 genome.

(**b**) Growth curves of *E. coli* ATCC 25922 upon 24 hr growth in Lysogeny Broth (LB) medium supplemented with mitomycin C (0 or 0.5 μg/mL) (n =3).

(**c**) PCR analysis to determine phage DNA fragments extracted from induced phage particles after 24 hr incubation. Lane 1: Positive control of phage Φ1 DNA fragment using bacterial genome as the template; Lane 2: Detection of phage Φ1 DNA fragment extracted from induced phage particles; Lane 3: Positive control of phage Φ2 DNA fragment using bacterial genome as the template; Lane 4: Detection of phage Φ2 DNA fragment extracted from induced phage particles; Lane 5: Positive control of phage Φ3 DNA fragment using bacterial genome as the template; Lane 6: Detection of phage Φ3 DNA fragment extracted from induced phage particles; Lane 7: Positive control of phage Φ4 DNA fragment using bacterial genome as the template; Lane 8: Detection of phage Φ4 DNA fragment extracted from induced phage particles; Lane 9: Positive control of phage Φ5 DNA fragment using bacterial genome as the template; Lane 10: Detection of phage Φ5 DNA fragment extracted from induced phage particles. The gel was derived from the same experiment and processed in parallel.

(**d**) Morphological analysis of phage-like particles by transmission electron microscopy.

Data in Supplementary Fig. 1b were analyzed using unpaired Student's t-test. ***$p < 0.001$. Data were expressed as mean ± SD.

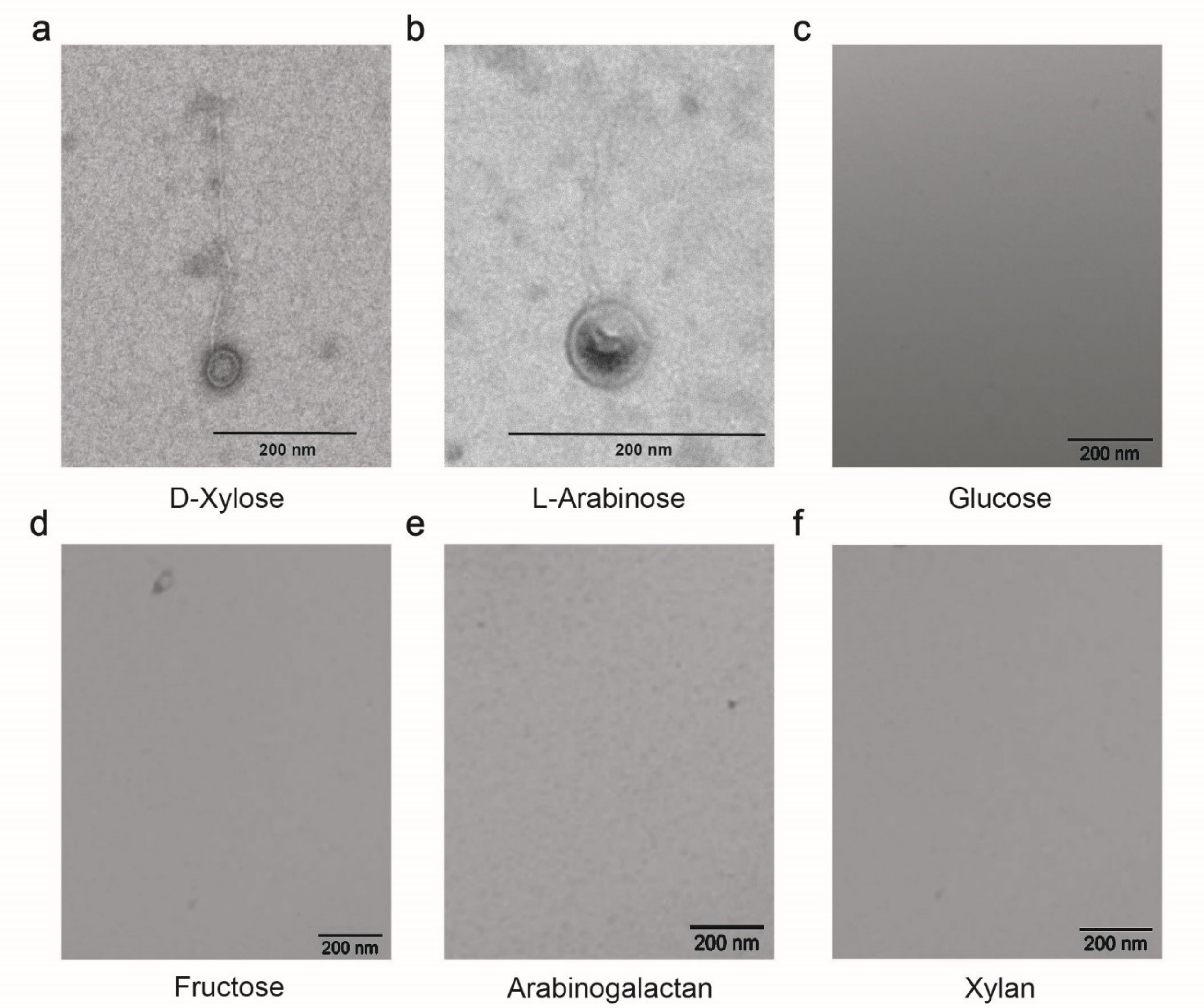

Supplementary Fig. 2 Phage-like particles in the culture supernatant under transmission electron microscopy

Observation of phage-like particles upon 24 hr growth in the culture medium supplemented with different carbon sources. (**a**) D-Xylose. (**b**) L-Arabinose. (**c**) Glucose. (**d**) Fructose. (**e**) Arabinogalactan. (**f**) Xylan.

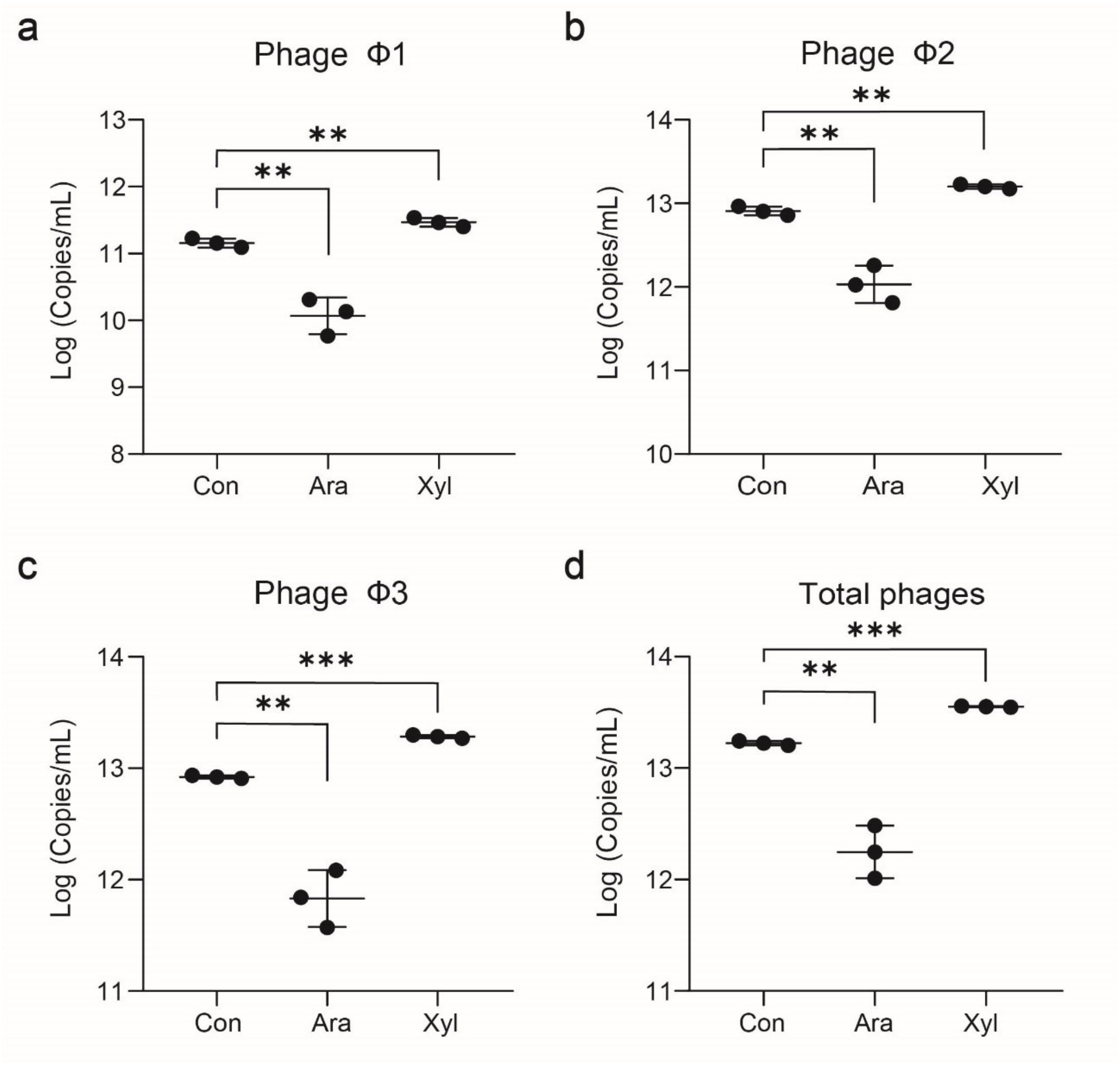

Supplementary Fig. 3 D-Xylose promotes phage production in *E. coli* ATCC 25922 *in vitro*

Phage Φ1 (**a**), Φ2 (**b**), Φ3 (**c**), and total phage production (sum of phage Φ1, Φ2, and Φ3) (**d**) of *E. coli* ATCC 25922 upon 24 hr growth in LB medium supplemented with 1% L-arabinose (Ara) or D-xylose (Xyl).

All data were analyzed using unpaired Student's t-test. $^{**}p < 0.01$, $^{***}p < 0.001$. Data were expressed as mean ± SD.

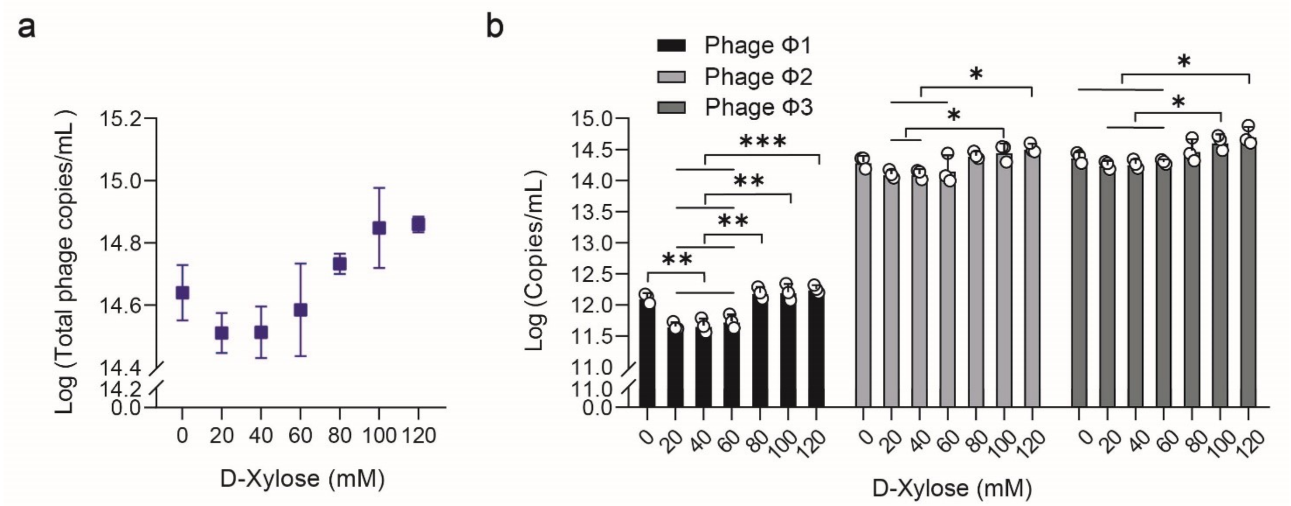

Supplementary Fig. 4 Phage production of *E. coli* ATCC 25922 upon 24 hr growth in MOPS minimal medium supplemented with 0, 20, 40, 60, 80, 100, and 120 mM D-xylose

(**a**) Total phage production (sum of phage Φ1, Φ2, and Φ3) (n = 3).

(**b**) Phage Φ1, Φ2, and Φ3 production (n = 3).

Data in Supplementary Fig. 4b were analyzed using one-way ANOVA with Tukey's test. *$p < 0.05$, **$p < 0.01$, ***$p < 0.001$. Data were expressed as mean ± SD.

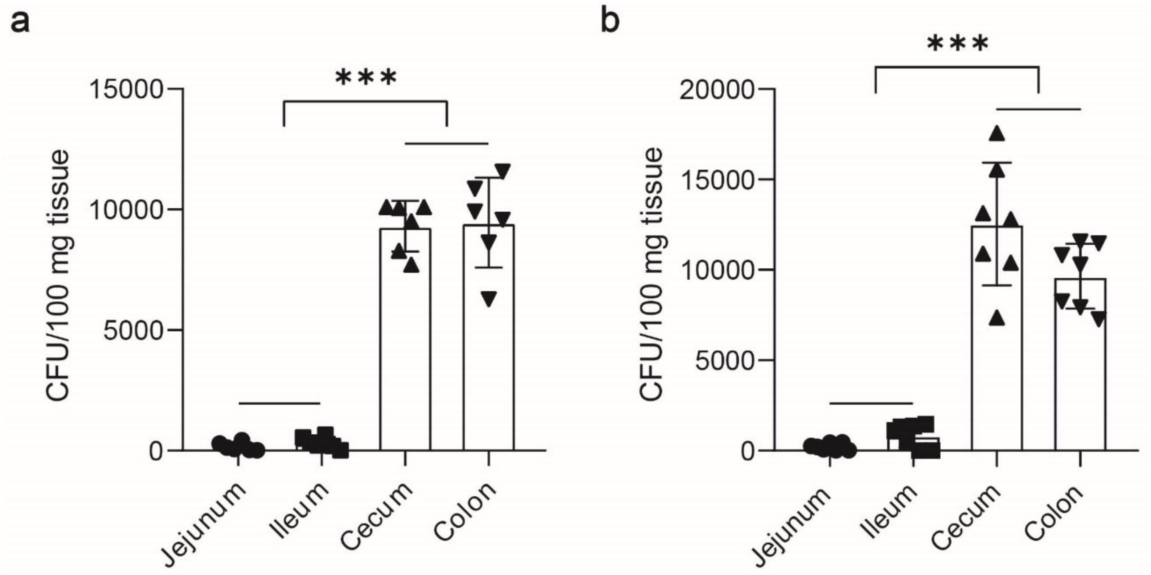

Supplementary Fig. 5 *E. coli* ATCC 25922 mainly colonizes the large intestine

(**a**) *E. coli* ATCC 25922 number in intestinal tissues after D-xylose intervention.

(**b**) *E. coli* ATCC 25922 number in intestinal tissues after sodium propionate intervention.

Six/seven mice were used for each treatment group. Each dot represents a single data point from a single mouse. All data were analyzed using one-way ANOVA with Tukey's test. ***$p < 0.001$. Data were expressed as mean ± SD.

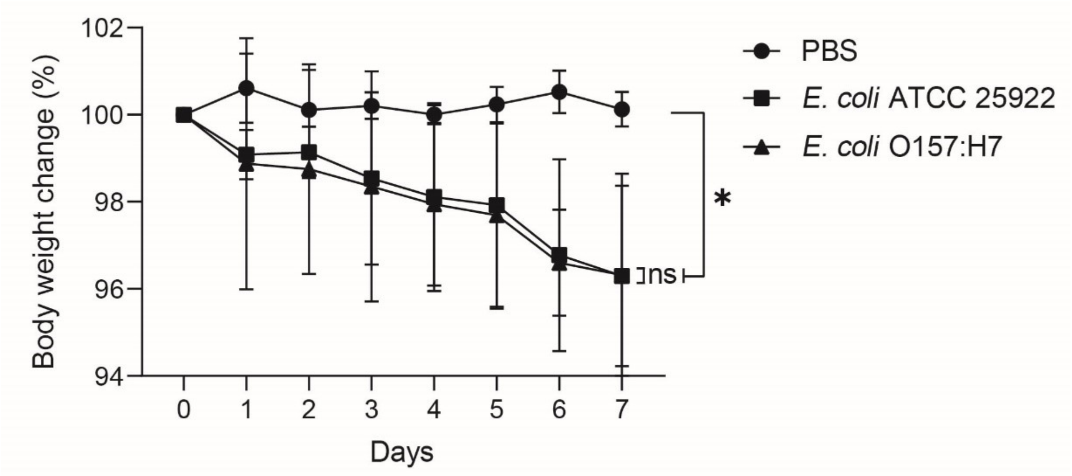

Supplementary Fig. 6 Body weight changes of mice daily infected with 5×10^7 CFU *E. coli* ATCC 25922 or *E. coli* O157:H7

Each dot represents the mean value of 6 mice per group daily. All data were analyzed using one-way ANOVA with Tukey's test. ns: not statistically significant. *$p < 0.05$. Data were expressed as mean ± SD.

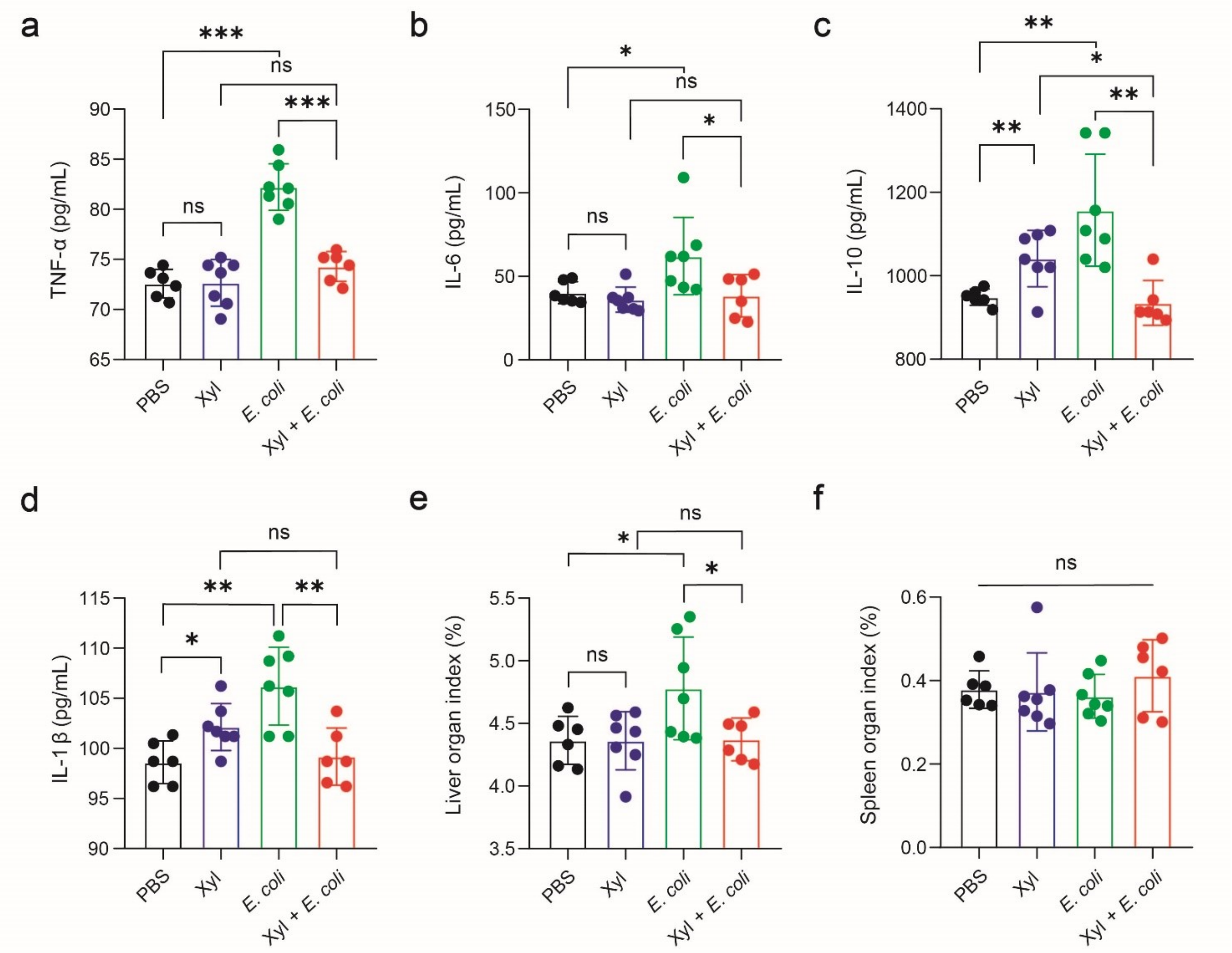

Supplementary Fig. 7 Serum levels of inflammatory factors and immune organ indexes in D-xylose (Xyl) intervention study

Levels of TNF-α (**a**), IL-6 (**b**), IL-10 (**c**), and IL-1β (**d**) in serum.

Liver organ index (**e**) and spleen organ index (**f**).

Six/seven mice were used for each treatment group. Each dot represents a single data point from a single mouse. All data were analyzed using unpaired Student's t-test. ns: not statistically significant. *$p < 0.05$, **$p < 0.01$, ***$p < 0.001$. Data were expressed as mean ± SD.

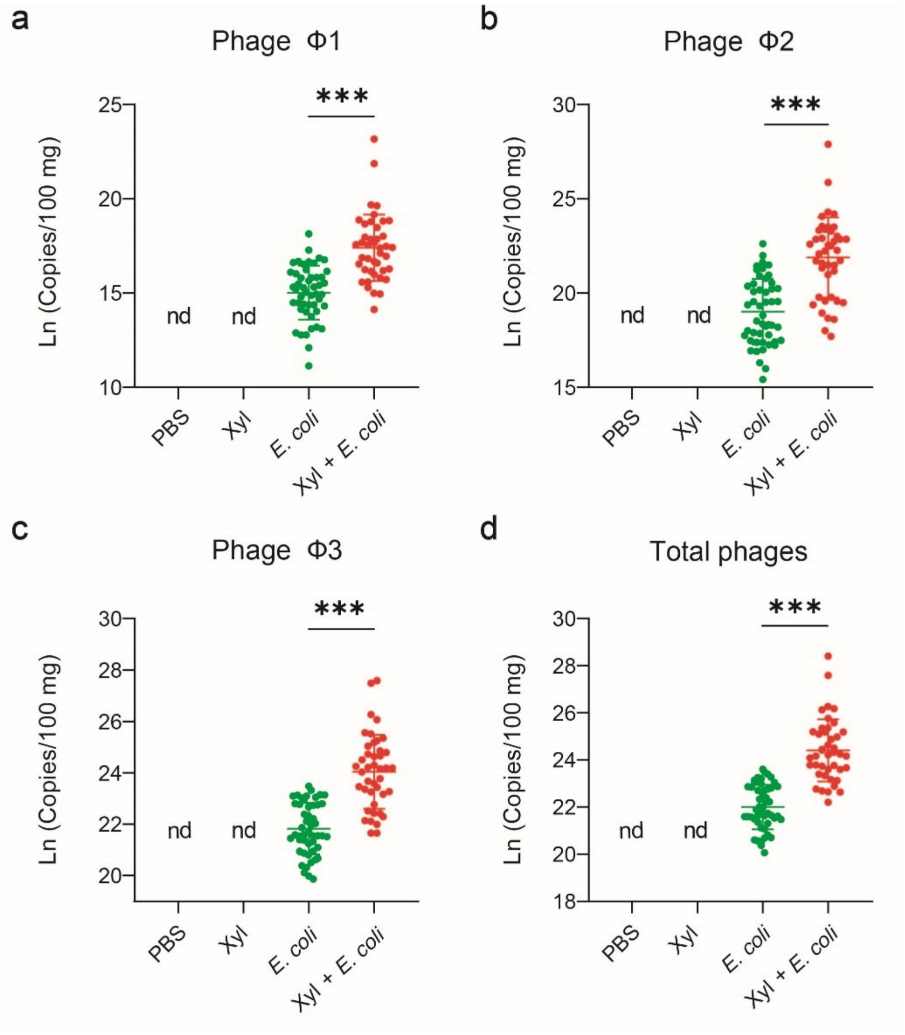

Supplementary Fig. 8 D-Xylose promotes phage production in *E. coli* ATCC 25922 *in vivo*

Number of phage Φ1 (**a**), Φ2 (**b**), Φ3 (**c**), and total phages (sum of phage Φ1, Φ2, and Φ3) (**d**) in feces during an experimental period of 7 days in D-xylose (Xyl) intervention study.

Six/seven mice were used for each treatment group. Each dot represents a single data point from a single mouse fecal sample on one day. nd: not detectable. All data were analyzed using unpaired Student's t-test. ***p < 0.001. Data were expressed as mean ± SD.

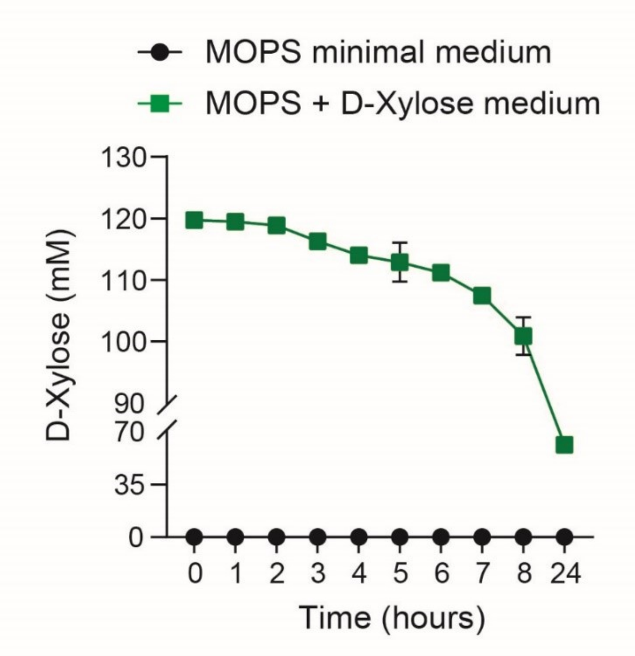

Supplementary Fig. 9 ***E. coli*** **ATCC 25922 metabolizes D-xylose**

Concentrations of D-xylose in MOPS minimal medium supplemented with 0 or 120 mM D-xylose (n = 3).

Data were expressed as mean ± SD.

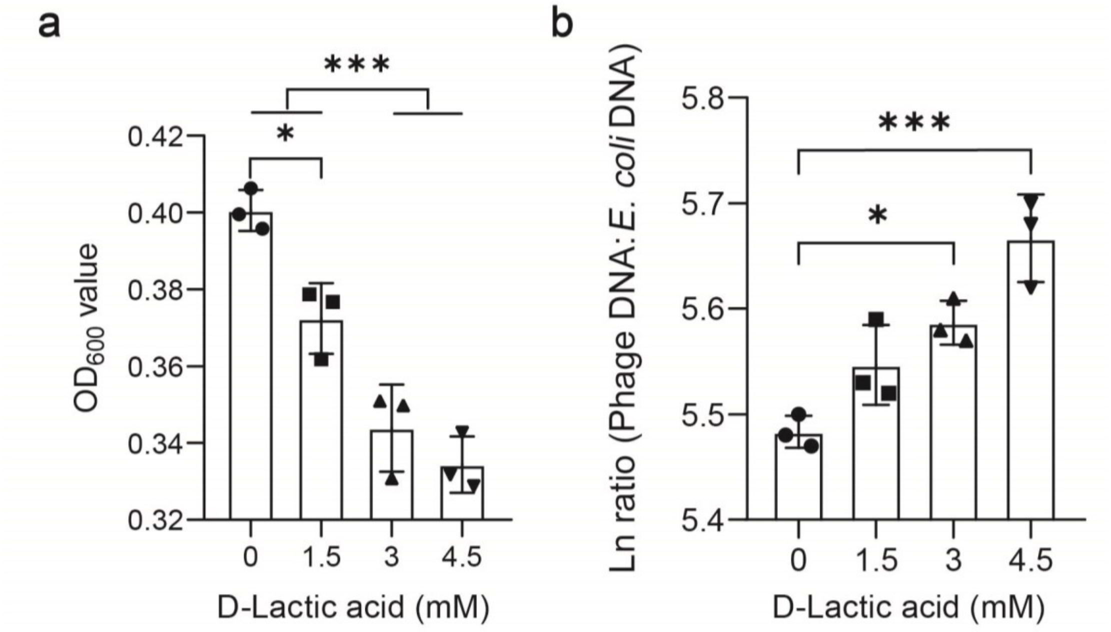

Supplementary Fig. 10 D-Lactic acid promotes prophage induction in *E. coli* ATCC 25922

E. coli ATCC 25922 cell density (**a**) and total phage (sum of phage Φ1, Φ2, and Φ3):*E. coli* DNA ratio (/mL) (**b**) upon 24 hr growth in MOPS minimal medium supplemented with 20 mM glucose and 0, 1.5, 3, or 4.5 mM D-lactic acid (n = 3 respectively).

All data were analyzed using one-way ANOVA with Tukey's test. *$p < 0.05$, ***$p < 0.001$. Data were expressed as mean ± SD.

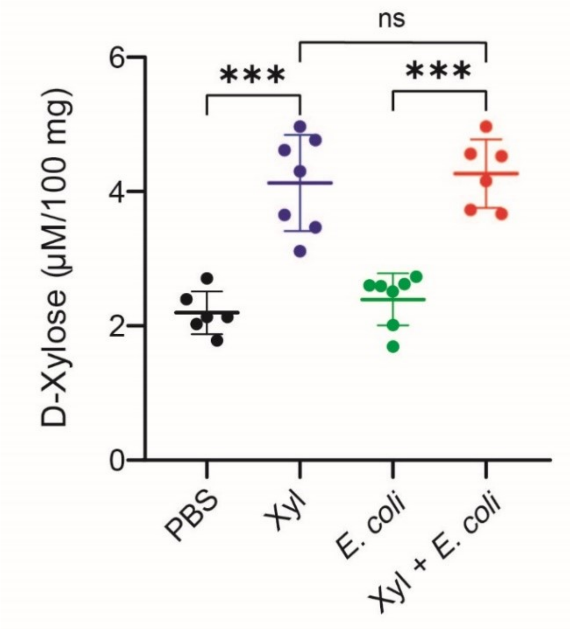

Supplementary Fig. 11 D-Xylose supplementation leads to D-xylose accumulation in the intestine of mice

Fecal D-xylose (xyl) concentrations on day 7 in D-xylose intervention study.

Six/seven mice were used for each treatment group. Each dot represents a single data point from a single mouse fecal sample. All data were analyzed using unpaired Student's t-test. ns: not statistically significant. ***$p < 0.001$. Data were expressed as mean ± SD.

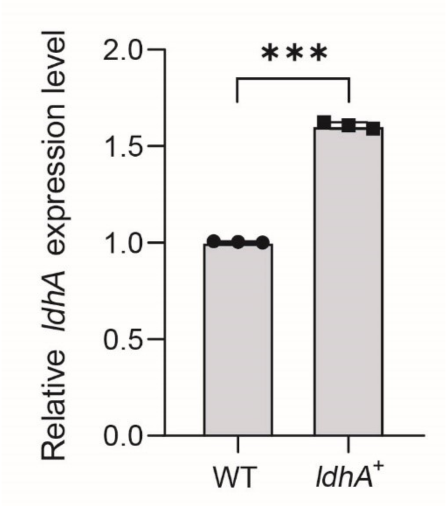

Supplementary Fig. 12 The *ldhA*$^{+}$ mutant of *E. coli* ATCC 25922 exhibits an increased gene expression level of *ldhA*

Relative *ldhA* expression levels of *E. coli* ATCC 25922 wild-type (WT) and *ldhA*-overexpressing (*ldhA*$^{+}$) strains upon 24 hr growth in MOPS minimal medium containing 100 mM D-xylose (n =3). Data were analyzed using unpaired Student's t-test. ***p < 0.001. Data were expressed as mean ± SD.

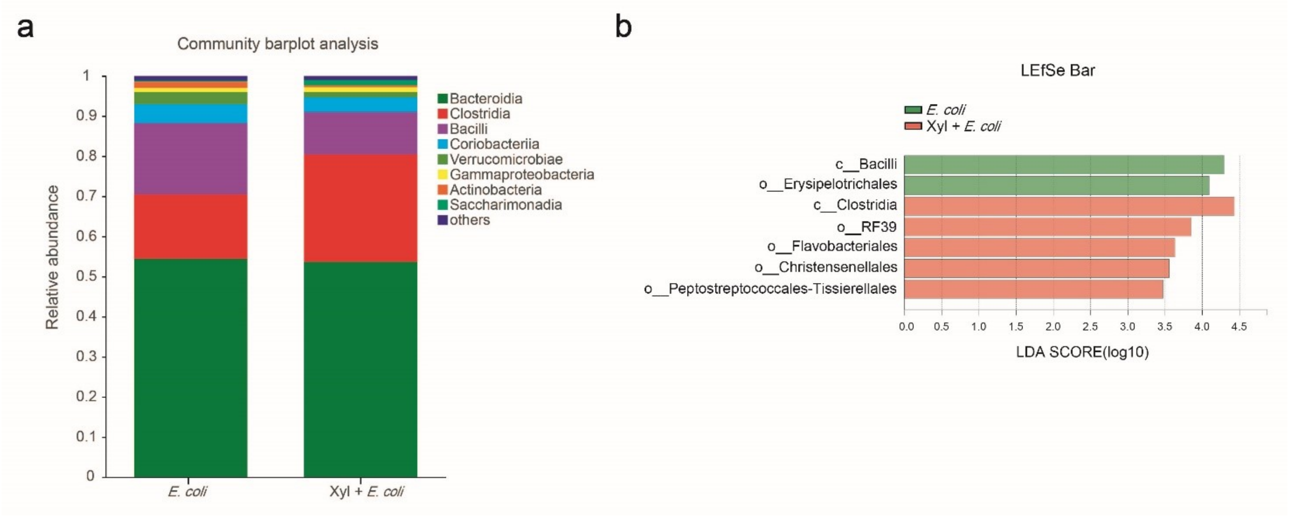

Supplementary Fig. 13 Clostridia is compositionally different microbiota between *E. coli* and D-xylose (Xyl) + *E. coli* treatment groups at the class level

(**a**) Fecal microbiota composition at the class level.

(**b**) LEfSe analysis of fecal microbiota composition at the class level.

Data represent the average microbial composition in fecal samples of 7 independent mice of each group.

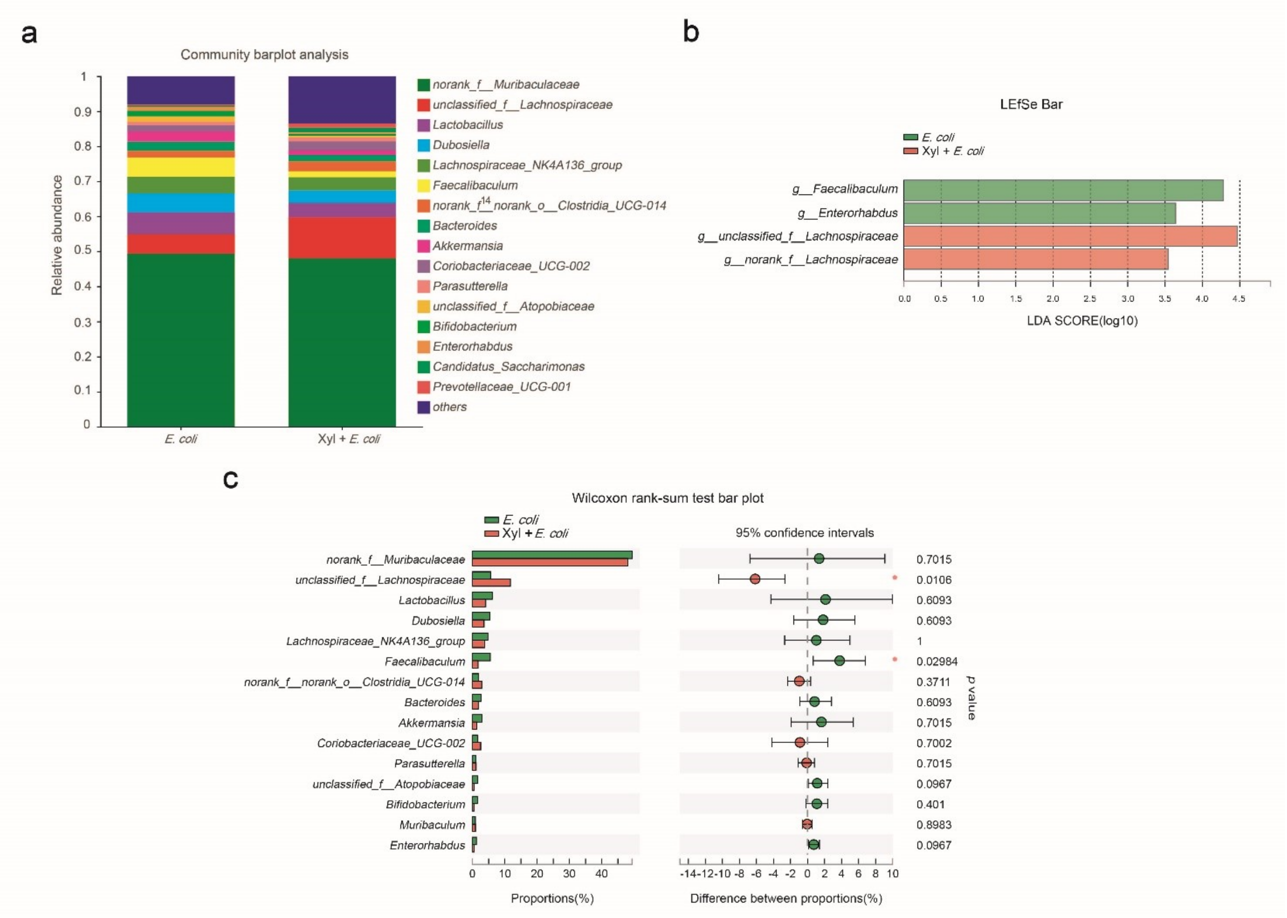

Supplementary Fig. 14 *Unclassified_f_Lachnospiraceae* is compositionally different microbiota between *E. coli* and D-xylose (Xyl) + *E. coli* treatment groups at the genus level

(**a**) Fecal microbiota composition at the genus level.

(**b**) LEfSe analysis of fecal microbiota composition at the genus level.

(**c**) Wilcoxon rank-sum test bar plot at the genus level.

Data represent the average microbial composition in fecal samples of 7 independent mice of each group.

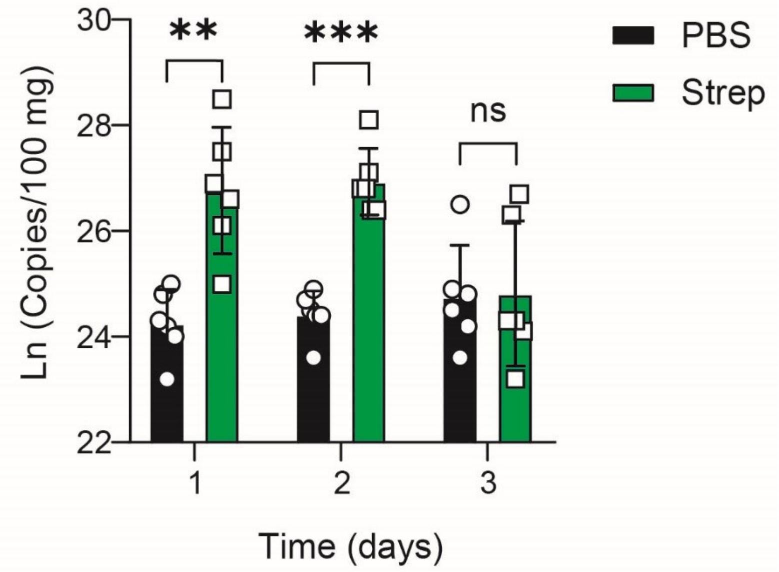

Supplementary Fig. 15 Fecal total phage production (sum of phage Φ1, Φ2, and Φ3) after streptomycin (Strep) treatment

Six mice were used for each treatment group. Each dot represents a single data point from a single mouse. All data were analyzed using unpaired Student's t-test. ns: not statistically significant. $^{**}p < 0.01$, $^{***}p < 0.001$. Data were expressed as mean ± SD.

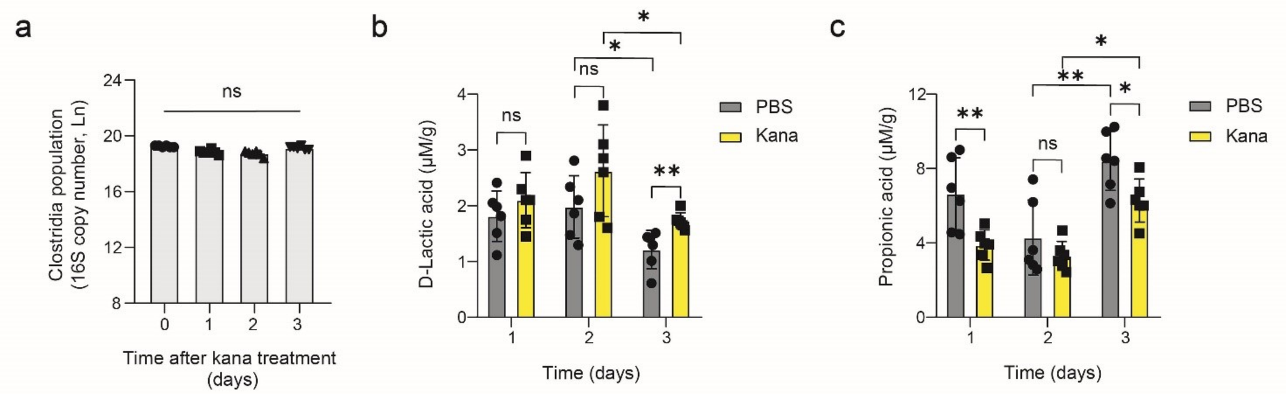

Supplementary Fig. 16 Gavage with kanamycin does not eliminate intestinal propionic acid and reduces the accumulation of D-lactic acid

(**a**) RT-qPCR analysis of fecal Clostridia copies after kanamycin (Kana) treatment.

Fecal D-lactic acid concentration (**b**) and propionic acid concentration (**c**).

Six mice were used for each treatment group. Each dot represents a single data point from a single mouse. Data in Supplementary Fig. 16a were analyzed using one-way ANOVA with Tukey's test. Data in Supplementary Fig. 16b, c were analyzed using unpaired Student's t-test. ns: not statistically significant. *$p < 0.05$, **$p < 0.01$. Data were expressed as mean ± SD.

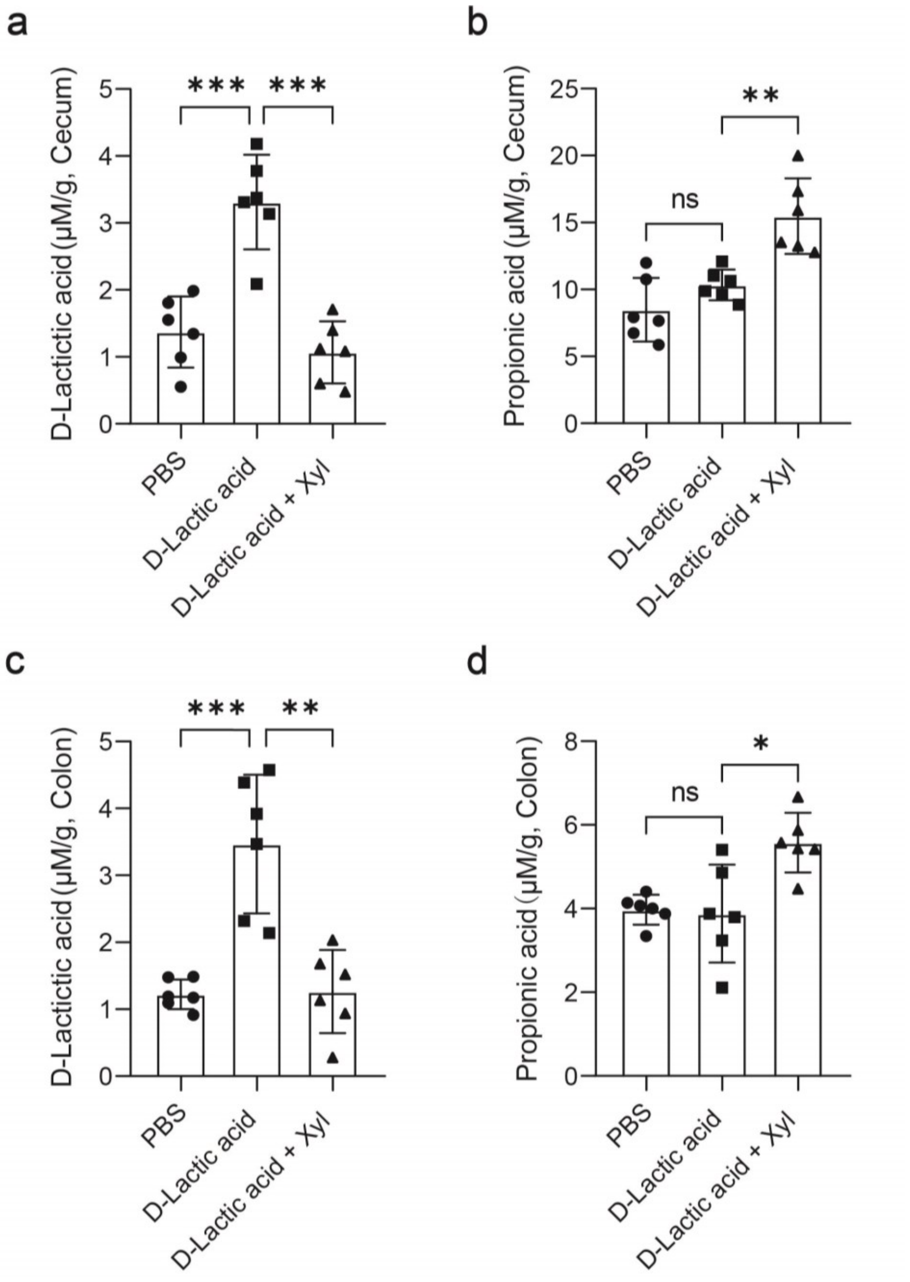

Supplementary Fig. 17 D-Xylose supplementation decreases intestinal D-lactic acid concentrations and increases propionic acid concentrations in mice gavaged with D-lactic acid

D-Lactic acid and propionic acid concentrations in cecum (**a**, **b**) and colon (**c**, **d**) digesta samples at 1.5 hr after mice orally administrated with D-lactic acid (10 mg).

Six mice were used for each treatment group. Each dot represents a single data point from a single mouse. Xyl: D-Xylose. All data were analyzed using unpaired Student's t-test. ns: not statistically significant. *$p < 0.05$, **$p < 0.01$, ***$p < 0.001$. Data were expressed as mean ± SD.

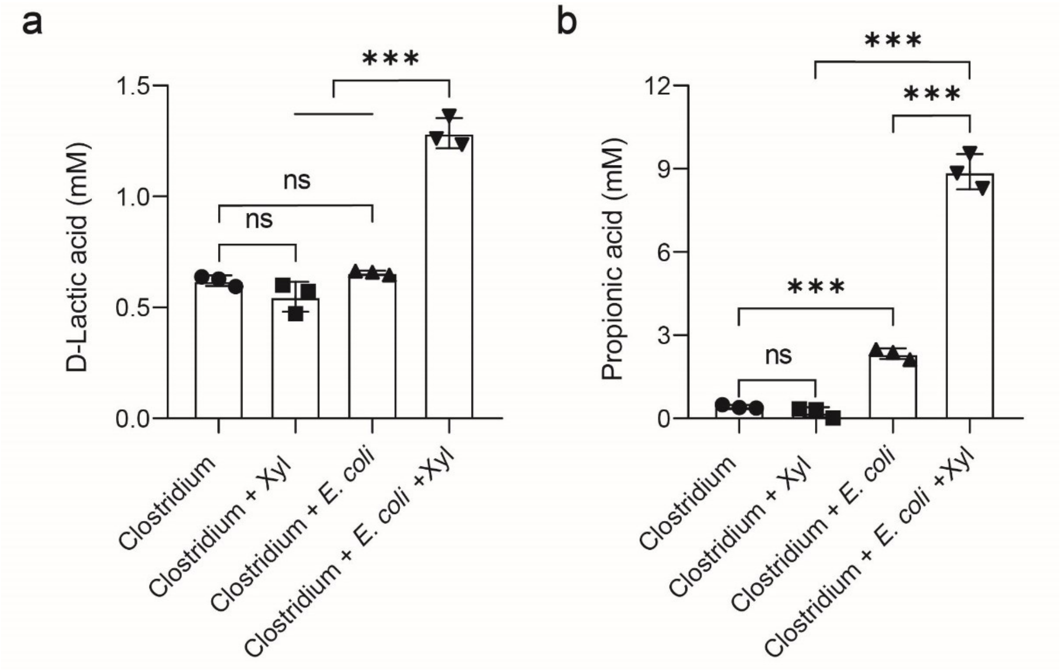

Supplementary Fig. 18 *E. coli* ATCC 25922-Clostridium symbiosum co-culture increases D-lactic acid and propionic acid production in medium containing D-xylose

D-Lactic acid (**a**) and propionic acid (**b**) concentrations in *E. coli* ATCC 25922-Clostridium symbiosum co-culture system (n =3 respectively).

All data were analyzed using unpaired Student's t-test. ns: not statistically significant. ***$p < 0.001$.

Data were expressed as mean ± SD. Xyl: D-Xylose (20 mM).

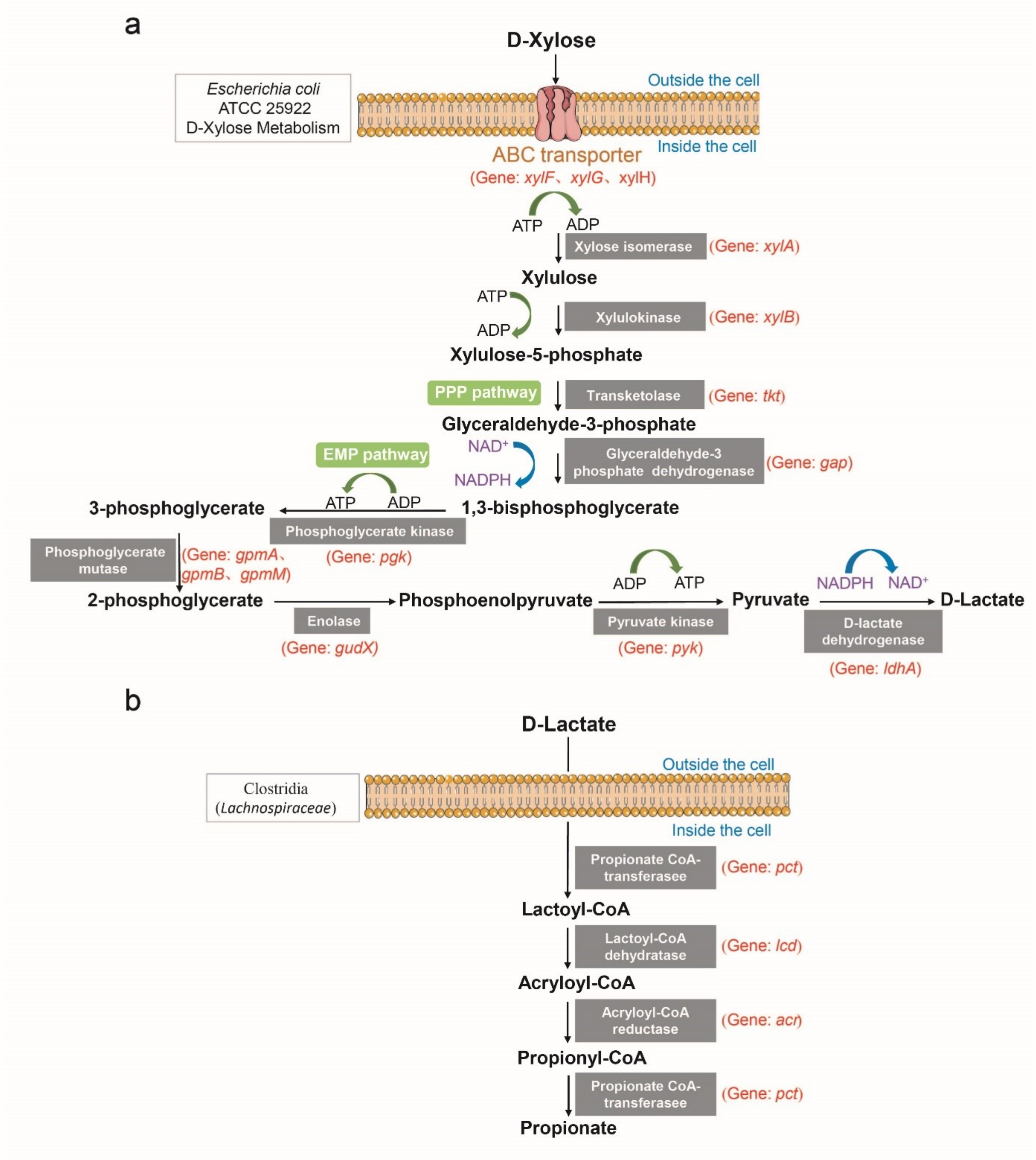

Supplementary Fig. 19 Predicted metabolic pathways from D-xylose to D-lactate and from D-lactate to propionate

(**a**) Predicted metabolic pathway from D-xylose to D-lactate in *E. coli* ATCC 25922.

(**b**) Predicted metabolic pathway from D-lactate to propionate in Clostridia (*Lachnospiraceae*).

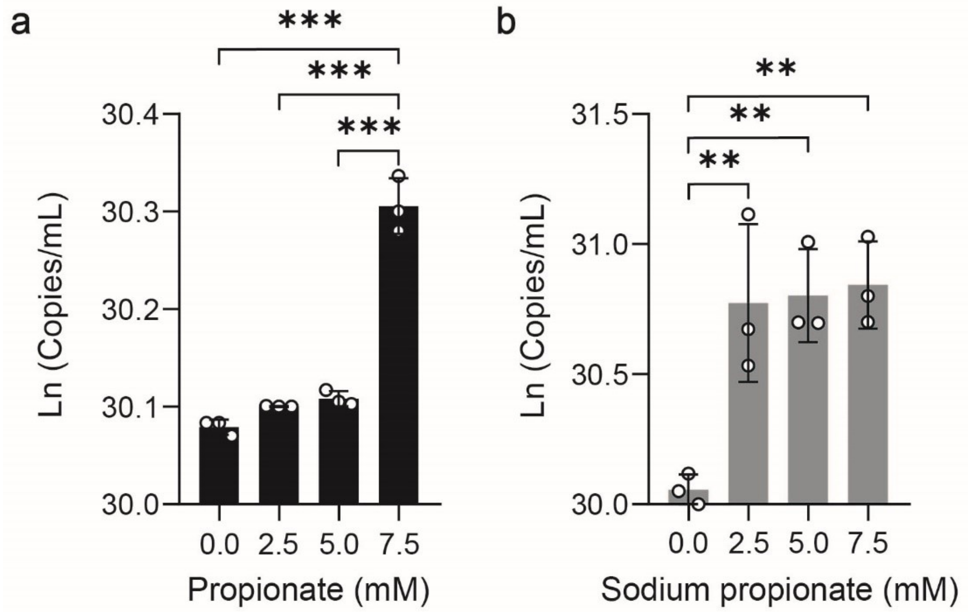

Supplementary Fig. 20 Propionate and sodium propionate promote phage production in *E. coli* ATCC 25922 *in vitro*

Total phage production (sum of phage Φ1, Φ2, and Φ3) of *E. coli* ATCC 25922 upon 24 hr growth in LB medium supplemented with 0, 2.5, 5.0, and 7.5 mM propionate (**a**) or sodium propionate (**b**) (n = 3 respectively).

All data were analyzed using one-way ANOVA with Tukey's test. ***p* < 0.01, ****p* < 0.001. Data were expressed as mean ± SD.

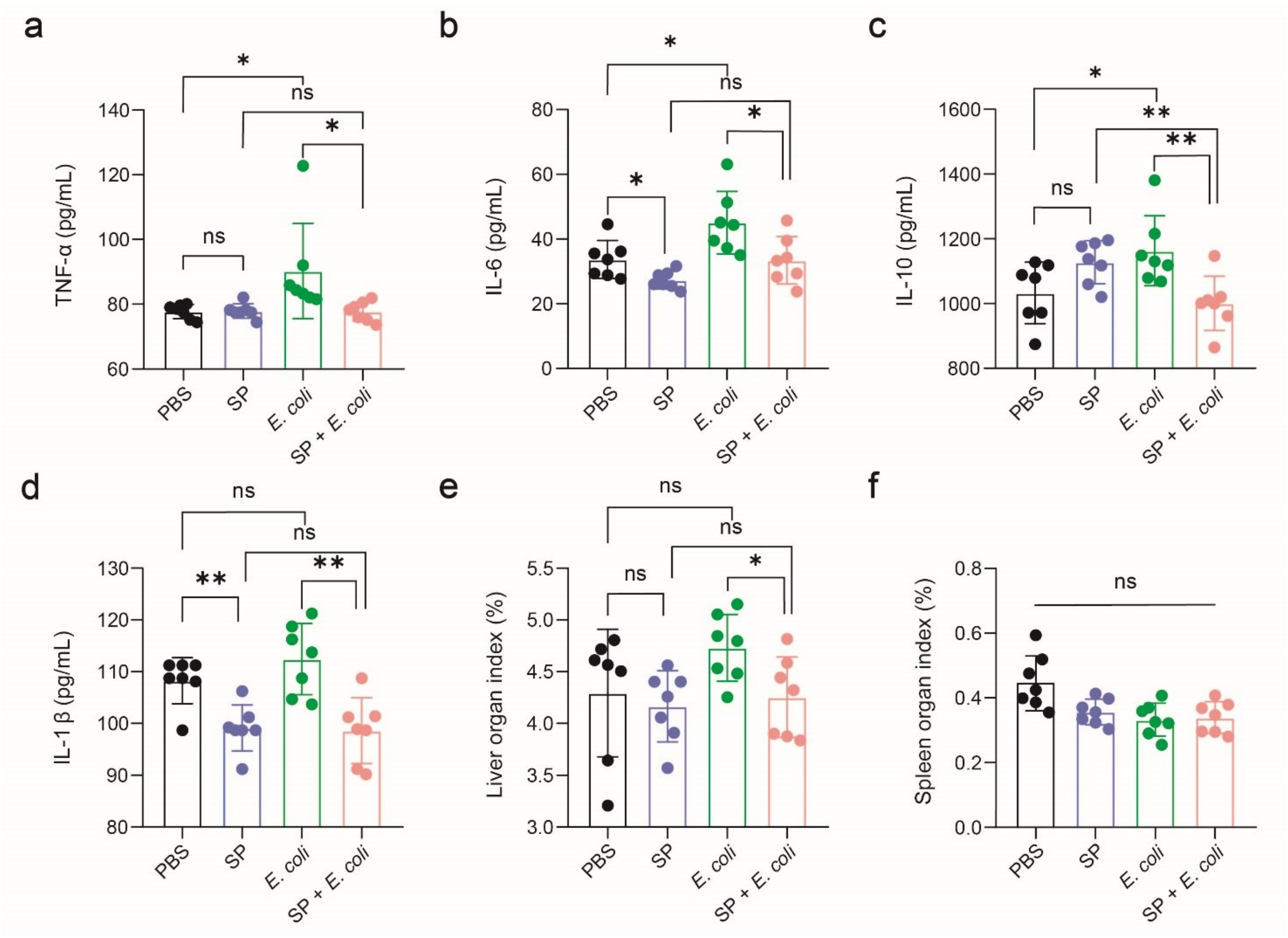

Supplementary Fig. 21 Serum levels of inflammatory factors and immune organ indexes in sodium propionate (SP) intervention study

Levels of TNF-α (**a**), IL-6 (**b**), IL-10 (**c**), and IL-1β (**d**) in serum.

Liver organ index (**e**) and spleen organ index (**f**).

Seven mice were used for each treatment group. Each dot represents a single data point from a single mouse. All data were analyzed using unpaired Student’s t-test. ns: not statistically significant. *p < 0.05, **p < 0.01. Data were expressed as mean ± SD.

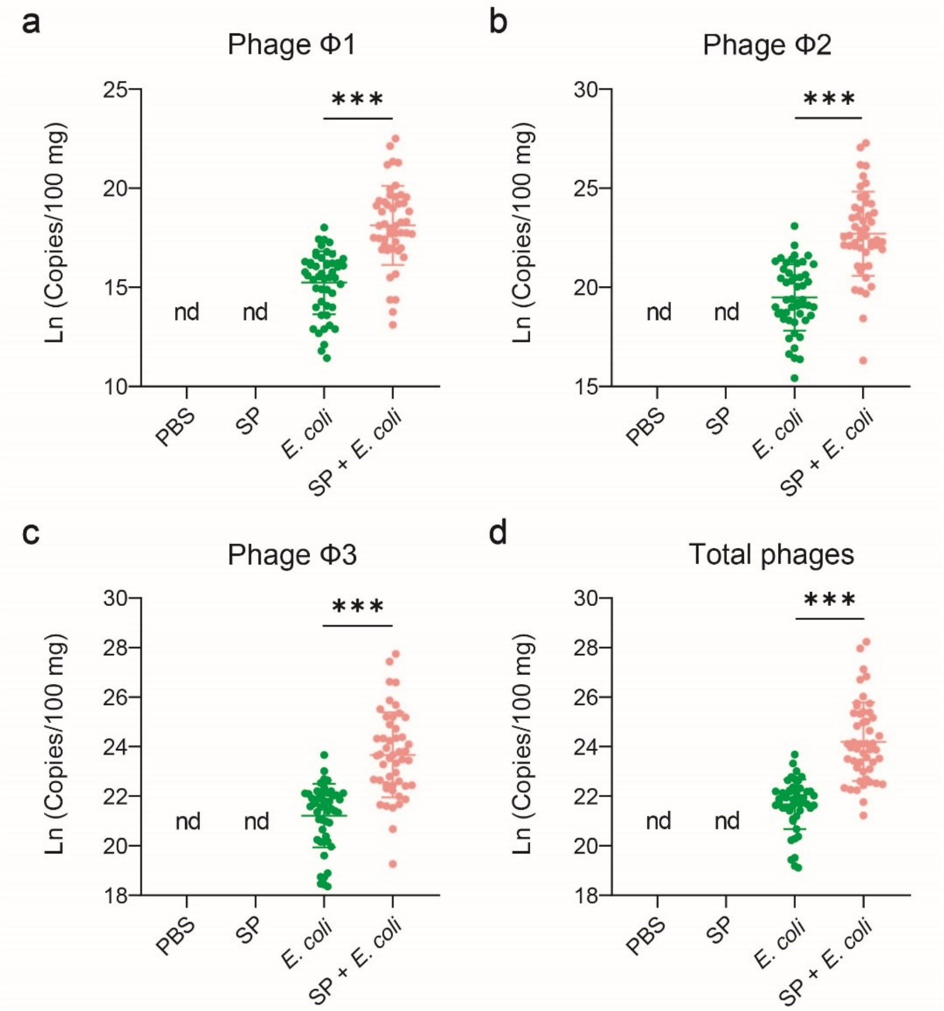

Supplementary Fig. 22 Sodium propionate promotes phage production in *E. coli* ATCC 25922 *in vivo*

Number of phage Φ1 (**a**), Φ2 (**b**), Φ3 (**c**), and total phages (sum of phage Φ1, Φ2, and Φ3) (**d**) in feces during an experimental period of 7 days in sodium propionate (SP) intervention study.

Seven mice were used for each treatment group. Each dot represents a single data point from a single mouse fecal sample on one day. nd: not detectable. All data were analyzed using unpaired Student's t-test. ***$p < 0.001$. Data were expressed as mean ± SD.

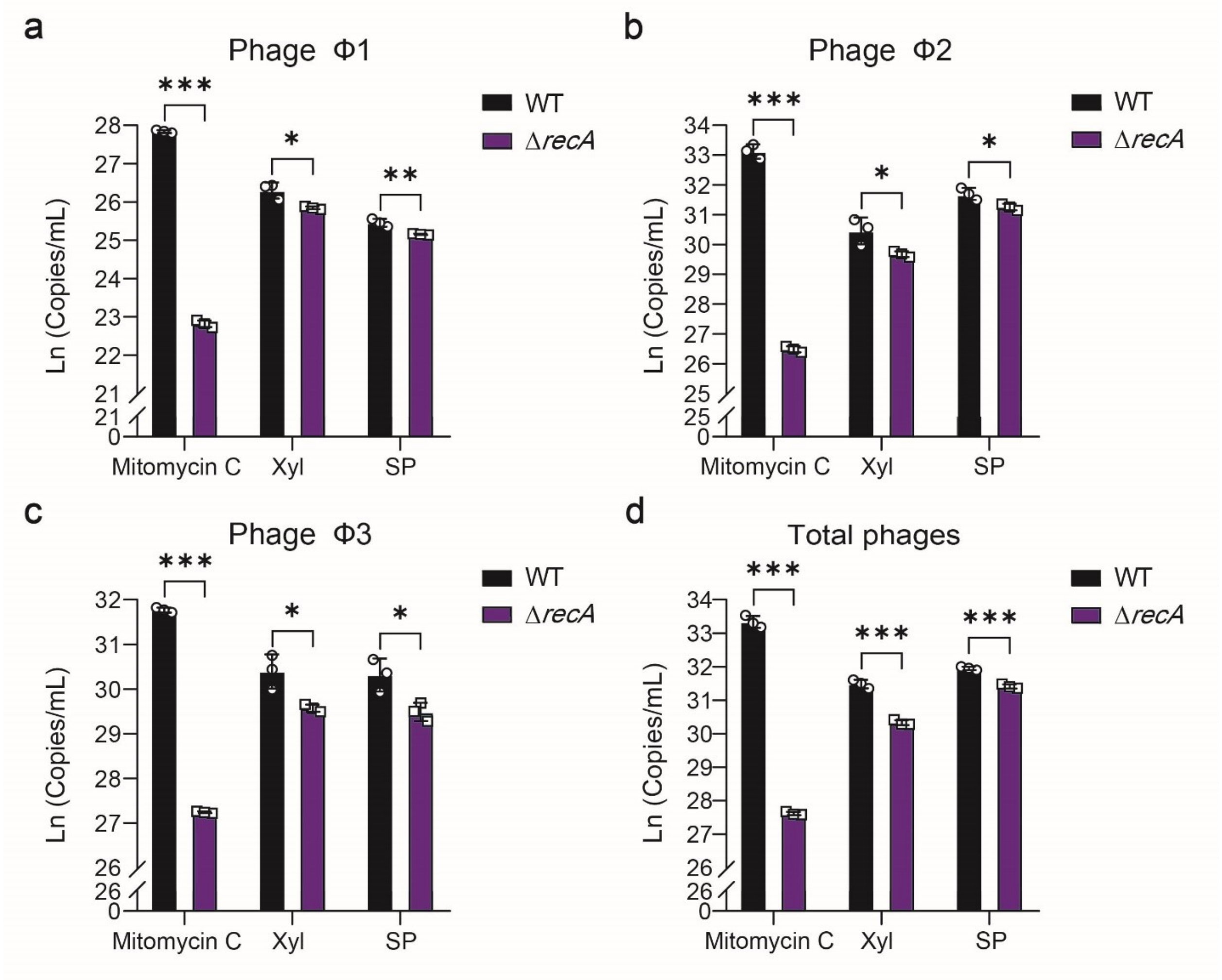

Supplementary Fig. 23 Knockout of *recA* reduces phage production in *E. coli* ATCC 25922 *in vitro*

Phage Φ1 (**a**), Φ2 (**b**), Φ3 (**c**), and total phage production (sum of phage Φ1, Φ2, and Φ3) (**d**) of *E. coli* ATCC 25922 wild-type (WT) and *recA*-deficient (Δ*recA*) strains in culture medium supplemented with mitomycin C (0.5 μg/mL), 100 mM D-xylose (Xyl), and 7.5 mM sodium propionate (SP) (n = 3 respectively).

All data were analyzed using unpaired Student's t-test. *$p < 0.05$, **$p < 0.01$, ***$p < 0.001$. Data were expressed as mean ± SD.

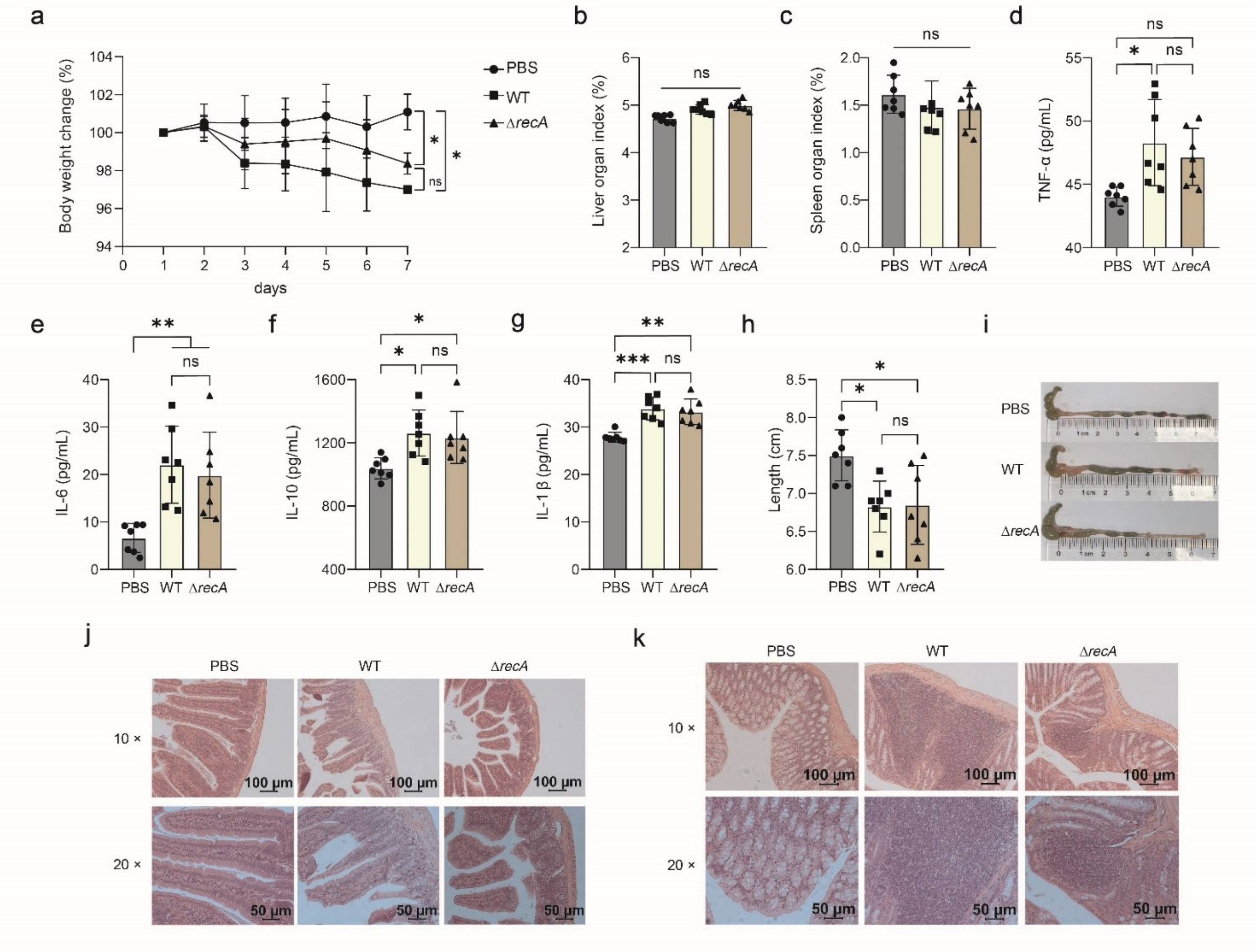

Supplementary Fig. 24 Knockout of *recA* does not affect pathogenicity of *E. coli* ATCC 25922

(**a**) Body weight change.

Liver organ index (**b**) and spleen organ index (**c**).

Levels of TNF-α (**d**), IL-6 (**e**), IL-10 (**f**), and IL-1β (**g**) in serum.

Colon length (**h**), representative colon images (**i**), and representative H&E staining images (Scale bars = 100 μm) of jejunum (**j**) and colon (**k**) and corresponding local high magnification images (Scale bars = 50 μm).

Seven mice were used for each treatment group. Each dot in Supplementary Fig. 24a represents the mean value of 7 mice per group daily. Each dot in Supplementary Fig. 24b-h represents a single data point from a single mouse. All data were analyzed using one-way ANOVA with Tukey's test. ns: not statistically significant. **p* < 0.05, ***p* < 0.01, ****p* < 0.001. Data were expressed as mean ± SD. WT: wild-type. Δ*recA*: *recA*-deficient type.

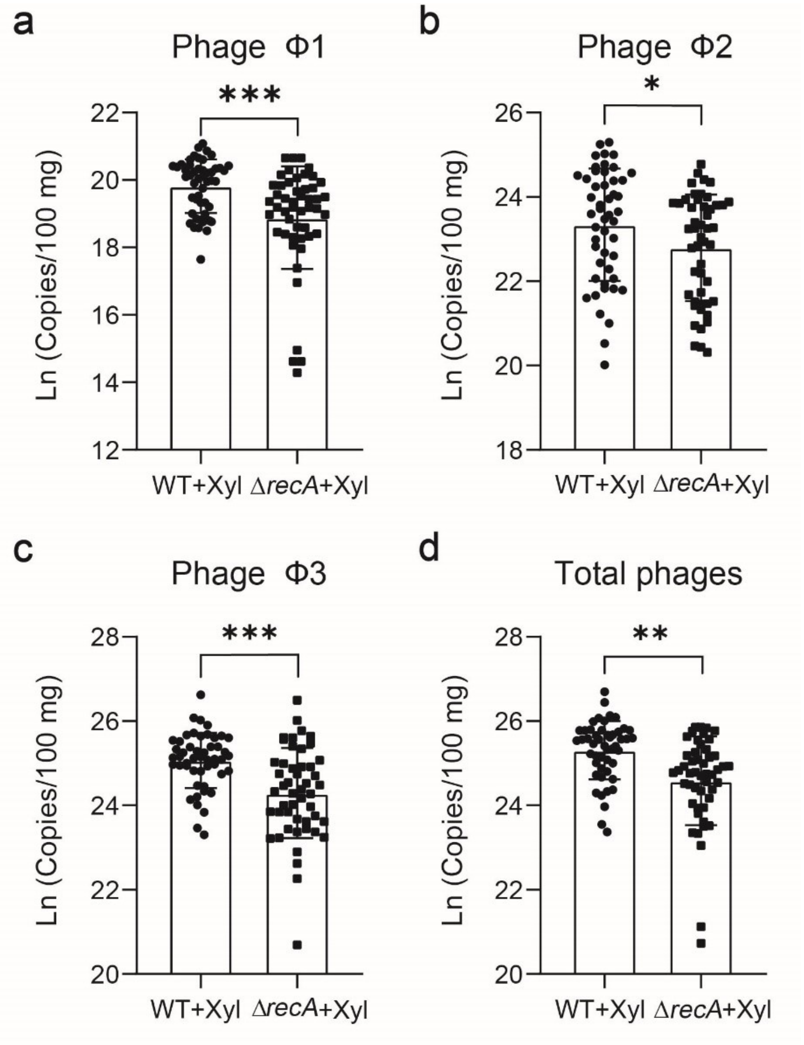

Supplementary Fig. 25 Knockout of *recA* reduces phage production in *E. coli* ATCC 25922 *in vivo*

Number of phage Φ1 (**a**), Φ2 (**b**), Φ3 (**c**), and total phages (sum of phage Φ1, Φ2, and Φ3) (**d**) in feces during an experimental period of 7 days.

Seven mice were used for each treatment group. Each dot represents a single data point from a single mouse fecal sample on one day. All data were analyzed using unpaired Student's t-test. $*p < 0.05$, $**p < 0.01$, $***p < 0.001$. Data were expressed as mean ± SD. WT: wild-type. Δ*recA*: *recA*-deficient type. Xyl: D-Xylose.

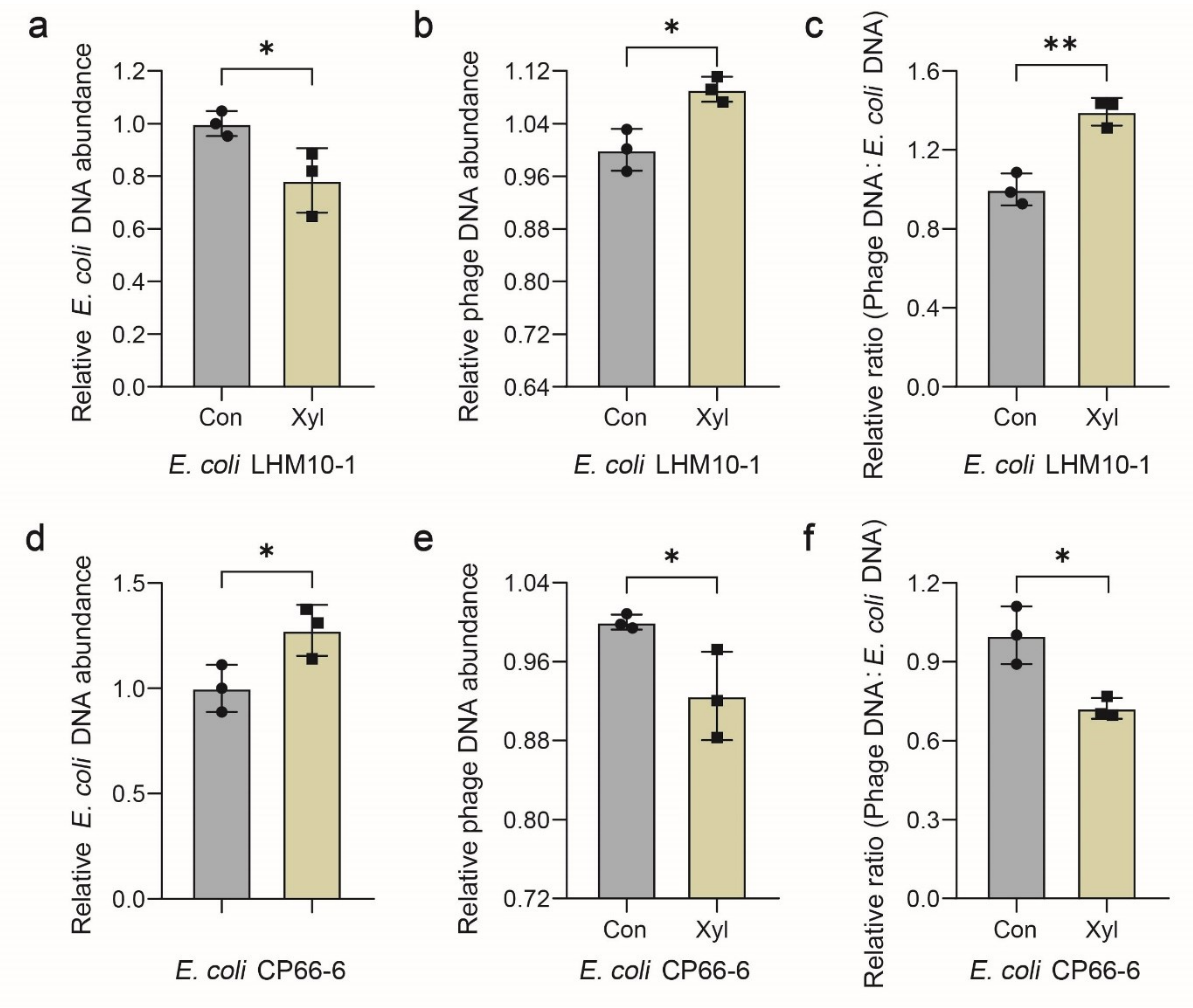

Supplementary Fig. 26 D-Xylose-mediated prophage induction in *E. coli* is strain-specific

Relative *E. coli* DNA abundance (**a**), relative total phage DNA abundance (**b**), and relative total phage:*E. coli* DNA ratio (**c**) (/mL) of *E. coli* LHM10-1 upon 24 hr growth in LB medium supplemented with 100 mM D-xylose (Xyl) (n = 3 respectively).

Relative *E. coli* DNA abundance (**d**), relative total phage DNA abundance (**e**), and relative total phage:*E. coli* DNA ratio (**f**) (/mL) of *E. coli* CP66-6 upon 24 hr growth in LB medium supplemented with 100 mM D-xylose (n = 3 respectively).

All data were analyzed using unpaired Student's t-test. *$p < 0.05$, **$p < 0.01$. Data were expressed as mean ± SD.

Supplementary Table 1 Details of prophages in *E. coli* ATCC 25922 genome

Prophage	Completeness	Size (Kb)	Score	Position	Most common phage	Type (Family)	Genetic structure
Φ1	Complete	38.2	150	3489917-3528128	PHAGE_Entero_mEp460_NC_019716(24)	*Siphoviridae*	Lysin, tail, envelope, terminase, portal, protease
Φ2	Complete	49.3	150	3891555-3940922	PHAGE_Entero_lambda_NC_001416(27)	*Siphoviridae*	Tail, head, capsid, envelope, transposase, integrase
Φ3	Complete	51.3	150	3949156-4000518	PHAGE_Entero_lambda_NC_001416(17)	*Siphoviridae*	Fiber, tail, head, capsid, envelope, lysis, integrase
Φ4	Incomplete	11.2	40	106749-118006	PHAGE_Entero_N15_NC_001901(2)	*Siphoviridae*	Integrase, capsid, head
Φ5	Incomplete	18.7	60	2914661-2933389	PHAGE_Stx2_c_1717_NC_011357(3)	*Pankowvirus*	Transposase

Supplementary Table 2 Primers used in this study

Items	Primers	Concentrations	Description
Prophage Φ1	Forward: 5´-CTTTCCCTTTACAATCGTGG-3´<br>Reverse: 5´-GTCGCTTCATACTCCTGTTCTG-3´	10 µM	PCR
Prophage Φ2	Forward: 5´-TGTAATGGAAGATGGGAAAT-3´<br>Reverse: 5´-CTGGAGGGCAAAGAAGAT-3´	10 µM	PCR
Prophage Φ3	Forward: 5´-GATAACGAGGACATCACCCA-3´<br>Reverse: 5´-CATCCAGCAACAACAGACG-3´	10 µM	PCR
Prophage Φ4	Forward: 5´- GTTGCTTTCACCGTCCAGT-3´<br>Reverse: 5´-GCTGCCTCGATAATCTTGCT-3´	10 µM	PCR
Prophage Φ5	Forward: 5´-ACTTGTAAACTTTGCTGCTT-3´<br>Reverse: 5´-TTCTTCCGCACGATTTGTCC-3´	10 µM	PCR
Prophage Φ1	Forward: 5´-TTACGTTCTGACATAGGCTT-3´<br>Reverse: 5´-TAGTTTTGACAGCACACGTT-3´	10 µM	RT-qPCR
Prophage Φ2	Forward: 5´-AACCAATGCCTAACCCTCT-3´<br>Reverse: 5´-CGAAATCAGCACGAACTCT-3´	10 µM	RT-qPCR
Prophage Φ3	Forward: 5´-GCGGATTTCGTCTTGTATG-3´<br>Reverse: 5´-CAGGGCGAGTTTGATTTG-3´	10 µM	RT-qPCR
E. coli ATCC 25922	Forward: 5´-TTACGTTCTGACATAGGCTT-3´<br>Reverse: 5´-TAGTTTTGACAGCACACGTT-3´	10 µM	RT-qPCR
Gene *recA*	Forward: 5´-CAACAGAACATATTGACTATCCGG TATTACCCGGCATGACAGGAGTAAAACATATG AATATCCTCCTTAGTTCCTATTC-3´<br>Reverse: 5´-GGGCCGCAGATGCGACCCTTGTGT ATCAAACAAGACGATTAAAAATCTTCGAGCTG CTTCGAAGTTCCTA-3´	10 µM	Construction of *recA* mutant
Gene *ldhA*	Forward: 5´-CGAGGGTTTTGGAGCGGCT-3´<br>Reverse: 5´-CGCTGTAGCGAACAGTCACT-3´	10 µM	PCR validation for mutant
Gene *recA*	Forward: 5´-TTGCAACGCCAACACCATCT-3´<br>Reverse: 5´-ATCGCTACCAGCCGTTGCGT-3´	10 µM	PCR validation for mutant

Prophages in *E. coli* LHM10-1	1.Forward: 5´-TAAACACCGCCCTTTGCAT-3´<br>Reverse: 5´-TTCTTTCGCTACTTCCGTGA-3´<br>2.Forward: 5´-ACAATACCAGGACTATCCGTA-3´<br>Reverse: 5´-CGTATTGCCCATTTATCGAC-3´<br>3.Forward: 5´-TTACAGCCTGGACCCTC-3´<br>Reverse: 5´-AACGCATTTATCAAGCACAC-3´	10 μM	RT-qPCR
E. coli LHM10-1	Forward: 5´-ACAATACCAGGACTATCCGTA-3´<br>Reverse: 5´-CGTATTGCCCATTTATCGAC-3´	10 μM	RT-qPCR
Prophages in *E. coli* CP66-6	1.Forward: 5´-CGTTCAGCACCATTACGAA-3´<br>Reverse: 5´-CGAAAATGAACCAGCAGTCC-3´<br>2.Forward: 5´-CCATACAATCGGCTTCGT-3´<br>Reverse: 5´-TAATCACCGAAACCAAACCC-3´	10 μM	RT-qPCR
E. coli CP66-6	Forward: 5´-CCATACAATCGGCTTCGT-3´<br>Reverse: 5´-TAATCACCGAAACCAAACCC-3´	10 μM	RT-qPCR
