# Supplementary material for: Dietary D-xylose promotes intestinal health by inducing phage production in Escherichia coli
Source: NPJ Biofilms Microbiomes. 2023 Oct 11;9:79. doi: 10.1038/s41522-023-00445-w (PMC10567762; doi:10.1038/s41522-023-00445-w)
Supplement: Supplementary file 2 — Reporting Summary [file 41522_2023_445_MOESM2_ESM.pdf]

nature portfolio

Corresponding author(s): Dandan Han

Last updated by author(s): Aug 9, 2023

Reporting Summary

Nature Portfolio wishes to improve the reproducibility of the work that we publish. This form provides structure for consistency and transparency in reporting. For further information on Nature Portfolio policies, see our Editorial Policies and the Editorial Policy Checklist.

Statistics

For all statistical analyses, confirm that the following items are present in the figure legend, table legend, main text, or Methods section.

n/a	Confirmed	
☐	☒	The exact sample size (n) for each experimental group/condition, given as a discrete number and unit of measurement
☐	☒	A statement on whether measurements were taken from distinct samples or whether the same sample was measured repeatedly
☐	☒	The statistical test(s) used AND whether they are one- or two-sided<br>*Only common tests should be described solely by name; describe more complex techniques in the Methods section.*
☐	☒	A description of all covariates tested
☐	☒	A description of any assumptions or corrections, such as tests of normality and adjustment for multiple comparisons
☐	☒	A full description of the statistical parameters including central tendency (e.g. means) or other basic estimates (e.g. regression coefficient) AND variation (e.g. standard deviation) or associated estimates of uncertainty (e.g. confidence intervals)
☐	☒	For null hypothesis testing, the test statistic (e.g. F, t, r) with confidence intervals, effect sizes, degrees of freedom and P value noted<br>*Give P values as exact values whenever suitable.*
☐	☒	For Bayesian analysis, information on the choice of priors and Markov chain Monte Carlo settings
☐	☒	For hierarchical and complex designs, identification of the appropriate level for tests and full reporting of outcomes
☐	☒	Estimates of effect sizes (e.g. Cohen's d, Pearson's r), indicating how they were calculated

Our web collection on statistics for biologists contains articles on many of the points above.

Software and code

Policy information about availability of computer code

Data collection	Our manuscript did not utilize custom algorithms or software.
Data analysis	Our manuscript did not utilize custom algorithms or software.

For manuscripts utilizing custom algorithms or software that are central to the research but not yet described in published literature, software must be made available to editors and reviewers. We strongly encourage code deposition in a community repository (e.g. GitHub). See the Nature Portfolio guidelines for submitting code & software for further information.

Data

Policy information about availability of data

All manuscripts must include a data availability statement. This statement should provide the following information, where applicable:

- Accession codes, unique identifiers, or web links for publicly available datasets
- A description of any restrictions on data availability
- For clinical datasets or third party data, please ensure that the statement adheres to our policy

The dataset containing Illumina MiSeq raw reads for fecal bacterial 16S rRNA gene sequencing analyzed in this study (Fig. 4a, b, Supplementary Fig. 13a, b, and Supplementary Fig. 14a-c) is available in the NCBI Sequence Read Archive (SRA) under BioProject PRJNA816313. All other data that support the findings of this study are available from the corresponding author upon reasonable request.

Research involving human participants, their data, or biological material

Policy information about studies with human participants or human data. See also policy information about sex, gender (identity/presentation), and sexual orientation and race, ethnicity and racism.

Reporting on sex and gender	Our research is not involved human participants.
Reporting on race, ethnicity, or other socially relevant groupings	Our research is not involved human participants.
Population characteristics	Our research is not involved human participants.
Recruitment	Our research is not involved human participants.
Ethics oversight	Our research is not involved human participants.

Note that full information on the approval of the study protocol must also be provided in the manuscript.

Field-specific reporting

Please select the one below that is the best fit for your research. If you are not sure, read the appropriate sections before making your selection.

☒ Life sciences ☐ Behavioural & social sciences ☐ Ecological, evolutionary & environmental sciences

For a reference copy of the document with all sections, see nature.com/documents/nr-reporting-summary-flat.pdf

Life sciences study design

All studies must disclose on these points even when the disclosure is negative.

Sample size	For our in vitro experiments, 3 independent samples were included. For our in vivo experiments, at least 6 independent samples were included. Sample sizes were determined according to published studies.
Data exclusions	No data were excluded.
Replication	All experimental findings could be successfully repeated.
Randomization	All mice were randomly allocated into different experimental groups.
Blinding	Investigators were blinded to group allocation during data collection and/or analysis.

Reporting for specific materials, systems and methods

We require information from authors about some types of materials, experimental systems and methods used in many studies. Here, indicate whether each material, system or method listed is relevant to your study. If you are not sure if a list item applies to your research, read the appropriate section before selecting a response.

Materials & experimental systems

n/a	Involved in the study	
☒	☐	Antibodies
☒	☐	Eukaryotic cell lines
☒	☐	Palaeontology and archaeology
☐	☒	Animals and other organisms
☒	☐	Clinical data
☒	☐	Dual use research of concern
☒	☐	Plants

Methods

n/a	Involved in the study	
☒	☐	ChIP-seq
☒	☐	Flow cytometry
☒	☐	MRI-based neuroimaging

Animals and other research organisms

Policy information about studies involving animals; ARRIVE guidelines recommended for reporting animal research, and Sex and Gender in Research

Laboratory animals	C57BL/6J mice were used in our study.
Wild animals	Wild animals were not used in our study.
Reporting on sex	Male C57BL/6J mice were used in our study. Therefore, our findings might only apply to male mice. Considering the physiological cycles in female mice, which may affect the accuracy of experimental results, we did not conduct experiments with female mice.
Field-collected samples	Six-week-old male C57BL/6J mice were used in this study. Maintenance feeds were provided for mice. All the mice had ad libitum access to water. Mice were housed under controlled conditions at 25°C, 55 ± 5% humidity, and 12-h light/dark cycle. At the end of experiments, mice were anaesthetized and euthanized by cervical dislocation. Spleen, liver, blood, and intestinal tissues were collected.
Ethics oversight	All animal studies were performed in accordance with the animal experimental ethics committee guidelines of China Agricultural University under protocol number AW21102202-1-1.

Note that full information on the approval of the study protocol must also be provided in the manuscript.
